# Elevated radium levels in Nubian Aquifer groundwater of Northeastern Africa

**DOI:** 10.1038/s41598-020-80160-0

**Published:** 2021-01-08

**Authors:** Mahmoud I. Sherif, Neil C. Sturchio

**Affiliations:** 1 Department of Earth Sciences, University of Delaware, Newark, DE 19716 USA; 2 Department of Geology, Tanta University, Tanta, 31527 Egypt

**Keywords:** Environmental impact, Hydrology

## Abstract

The Nubian Sandstone Aquifer System in Northeast Africa and the Middle East is a huge water resource of inestimable value to the population. However, natural radioactivity impairs groundwater quality throughout the aquifer posing a radiological health risk to millions of people. Here we present measurements of radium isotopes in Nubian Aquifer groundwater from population centers in the Western Desert of Egypt. Groundwater has ^226^Ra and ^228^Ra activities ranging from 0.01 to 2.11 and 0.03 to 2.31 Bq/L, respectively. Most activities (combined ^226^Ra + ^228^Ra) exceed U.S. EPA drinking water standards. The estimated annual radiation doses associated with ingestion of water having the highest measured Ra activities are up to 138 and 14 times the WHO-recommended maxima for infants and adults, respectively. Dissolved Ra activities are positively correlated with barium and negatively correlated with sulfate, while barite is approximately saturated. In contrast, Ra is uncorrelated with salinity. These observations indicate the dominant geochemical mechanisms controlling dissolved Ra activity may be barite precipitation and sulfate reduction, along with input from alpha-recoil and dissolution of aquifer minerals and loss by radioactive decay. Radium mitigation measures should be adopted for water quality management where Nubian Aquifer groundwater is produced for agricultural and domestic consumption.

## Introduction

**Figure 1:**
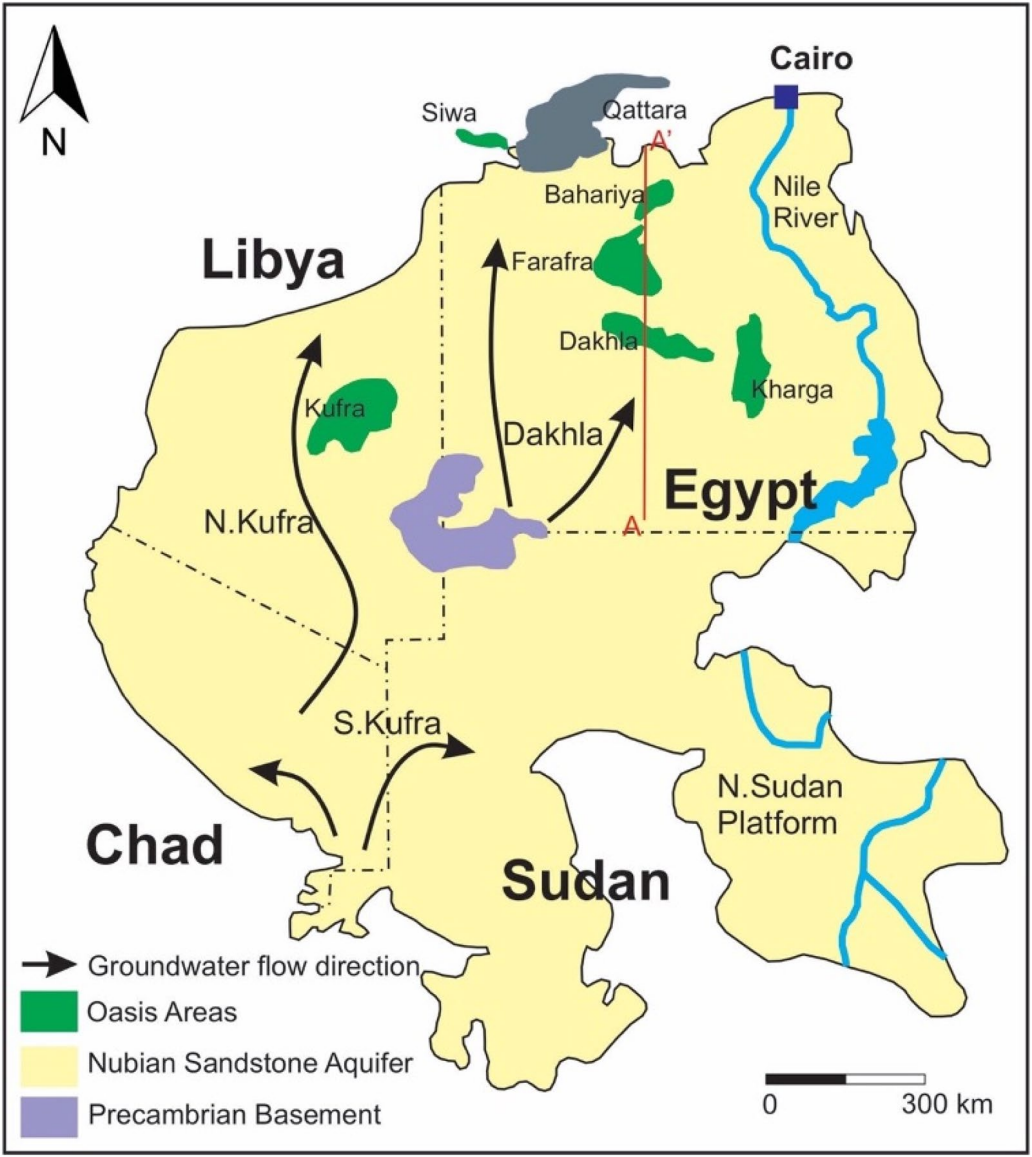
A map showing the areal extent of the Nubian Sandstone Aquifer System in northeast Africa, and the locations of the major subbasins and oasis areas (modified from^42^). Samples of this study were collected in Kharga, Dakhla, Farafra, Bahariya, and Siwa oases. Stratigraphic cross-section along line A–A′ is shown in Fig. 2.

Demand for freshwater in the Middle East and North Africa (MENA) region is increasing dramatically due to massive population growth, despite the scarcity of available freshwater resources. Freshwater per capita will be severely compromised as the region’s population is projected to increase. In 2018, the area had a population of 487.7 million people and as of 2017, its population increased by an annual average of 1.7%^1^. It is projected that the region’s population will reach 586 million by 2030 and 731 million by 2050^2^. The extensive groundwater reserves of the Nubian Sandstone Aquifer System (NSAS, henceforth referred to as Nubian Aquifer) provide a valuable resource to ameliorate water stress in several countries in the MENA region. The Nubian Aquifer is the world’s largest reservoir of fresh groundwater. It extends across four countries in northeast Africa (Egypt, Libya, Chad, and Sudan) as well as correlative aquifer formations in Israel, Jordan, Syria and the Arabian Peninsula covering a total area of nearly 2,000,000 square kilometers (Fig. 1). This region has a mostly hyper-arid climate with minimal potable surface water outside the Nile Valley.

Radium has been identified in several locations across the MENA region as a critical factor affecting the quality of groundwater supplies. Radium activities exceeding the maximum contaminant level (MCL) of drinking water have been reported in sandstone aquifer groundwaters from Jordan^3^, Israel^4,5^, Saudi Arabia^6^, and the Sinai Peninsula^7^ and Eastern Desert of Egypt^8^. However, there has not yet been a systematic study of radium in the three major Nubian aquifer subbasins of northeast Africa.

We measured the activities of the long-lived Ra isotopes, ^226^Ra (half-life = 1,600 a) and ^228^Ra (half-life = 5.75 a) in Nubian Aquifer groundwater from the Dakhla subbasin in the Western Desert of Egypt. We also investigated the geochemical mechanisms that control Ra behavior in this aquifer system and estimated the radiological dose rates to the populations that consume this groundwater. Findings of this study are of importance to water resource management in Egypt and are also of regional geopolitical significance because the Nubian Aquifer crosses a number of international boundaries.

### Regional hydrogeology

**Figure 2:**
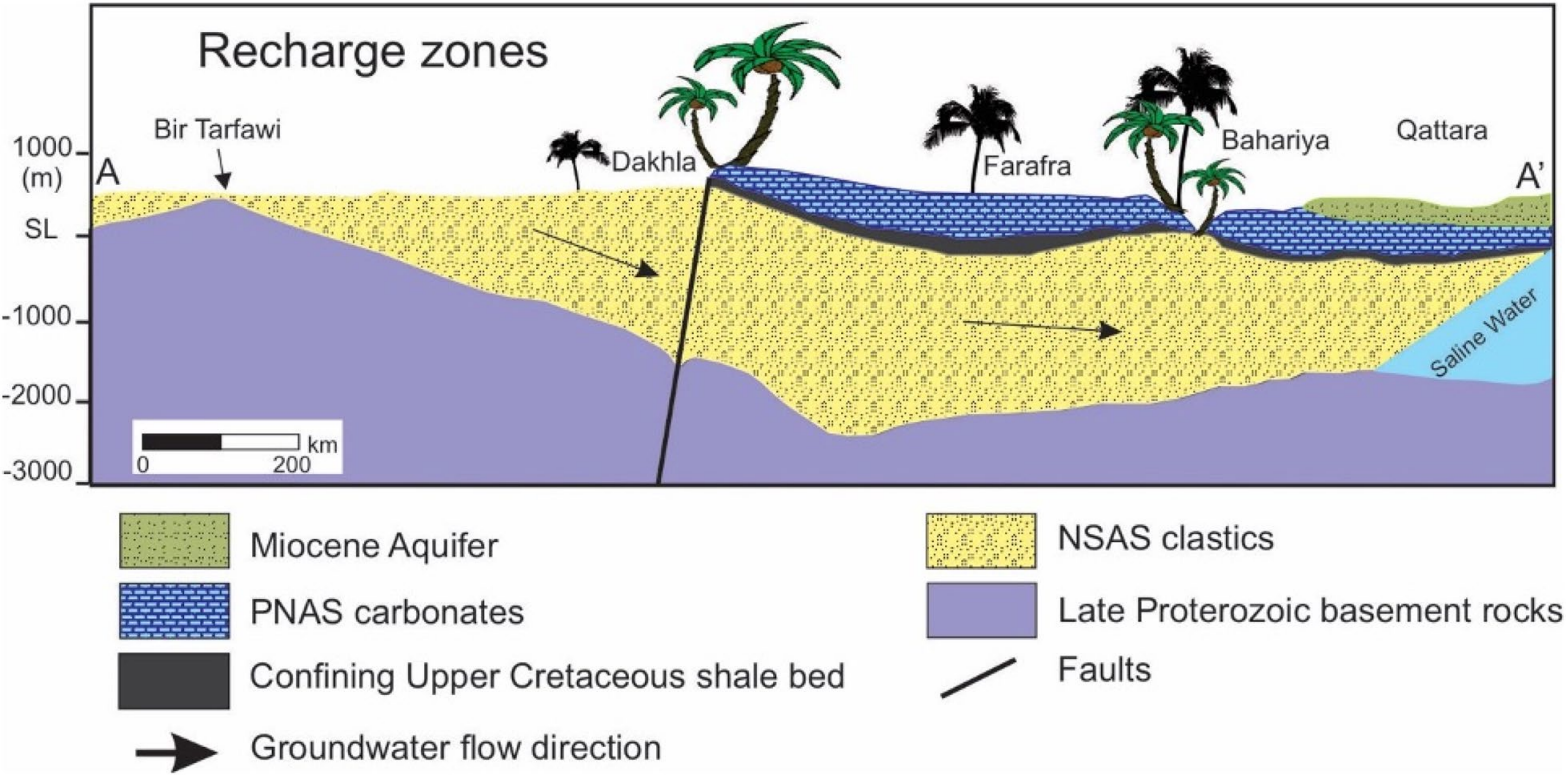
Simplified hydrostratigraphic cross-section along line A–A′ plotted in Fig. 1 showing the distribution of the Nubian Sandstone Aquifer System (modified from^42^). Recharge may occur in the unconfined portion of the aquifer upgradient of Dakhla. Elevations in meters above mean sea level. Palm trees not to scale.

The sedimentary formations that host the Nubian Aquifer formed after the Late Proterozoic times as a consequence of tectonic movements affecting the Arabian Nubian Shield. Tectonic stresses created regional uplifts and basins that were subsequently filled with continental sediments forming the host formations of the Nubian Aquifer. Figure 2 is a schematic cross-section showing the typical stratigraphic sequence of the Nubian Aquifer in the Western Desert of Egypt. The aquifer system includes three major subbasins: (1) Kufra subbasin (0.89 × 10^6^ km^2^) in Libya, northeastern Chad, and northwestern Sudan, (2) Dakhla subbasin (0.66 × 10^6^ km^2^) in Egypt, and (3) North Sudan Platform subbasin (0.36 × 10^6^ km^2^) in northern Sudan^9^ (Fig. 1). These subbasins are separated by northeastward and northwestward-trending basement uplifts. The Northern Sudan Platform subbasin is separated from the Dakhla subbasin to the north by the Uweinat-Aswan uplift and from the Kufra subbasin to the west by the Uweinat-Howar uplift^10^.

The Nubian Aquifer consists of highly porous clastic sediments of sandstone intercalated with clay beds, ranging from Cambrian to Upper Cretaceous. It lies uncomfortably on the rugged surface of the Proterozoic basement^11^. The Nubian Aquifer is overlain by the Post-Nubian Aquifer System (PNAS) which extends over North Eastern Libya and the northern part of the Western Desert of Egypt. The PNAS consists of marine sediments ranging in age from Upper Cenomanian to Holocene. The two aquifer systems are separated by low permeability confining layers of Upper Cretaceous to Lower Tertiary shales. However, connections between the two systems occur locally and are characterized by leakage between sedimentary sequences due to reduced thickness of the Upper Cretaceous-Lower Tertiary deposits or cross-cutting tectonic structures.

The Nubian Aquifer is extensively affected by deep fault systems trending E-W (Kalabsha trend) and NE-SW (Pelusium trend) in the southern and northern Western Desert, respectively^12,13^. Discharge of the Nubian Aquifer groundwater occurs largely as artesian leakage along faults that act as conduits for ascending groundwater in the lowlands of the Western Desert of Egypt (e.g., Farafra Oasis and the Qattara Depression) where the water table intersects the surface. Although the Nubian Aquifer provides a valuable resource of fresh groundwater, it is unsustainable due to low rainfall rate (≤ 5 mm yr^−1^), high evaporation rate, and minimal groundwater recharge in the region. Moreover, the large volume of groundwater extraction in populated areas is increasingly lowering the depth of the water table in populated oasis areas, ending discharge from former natural springs^14^.

## Results and discussion

### Radium occurrence and geochemical controls

**Figure 3:**
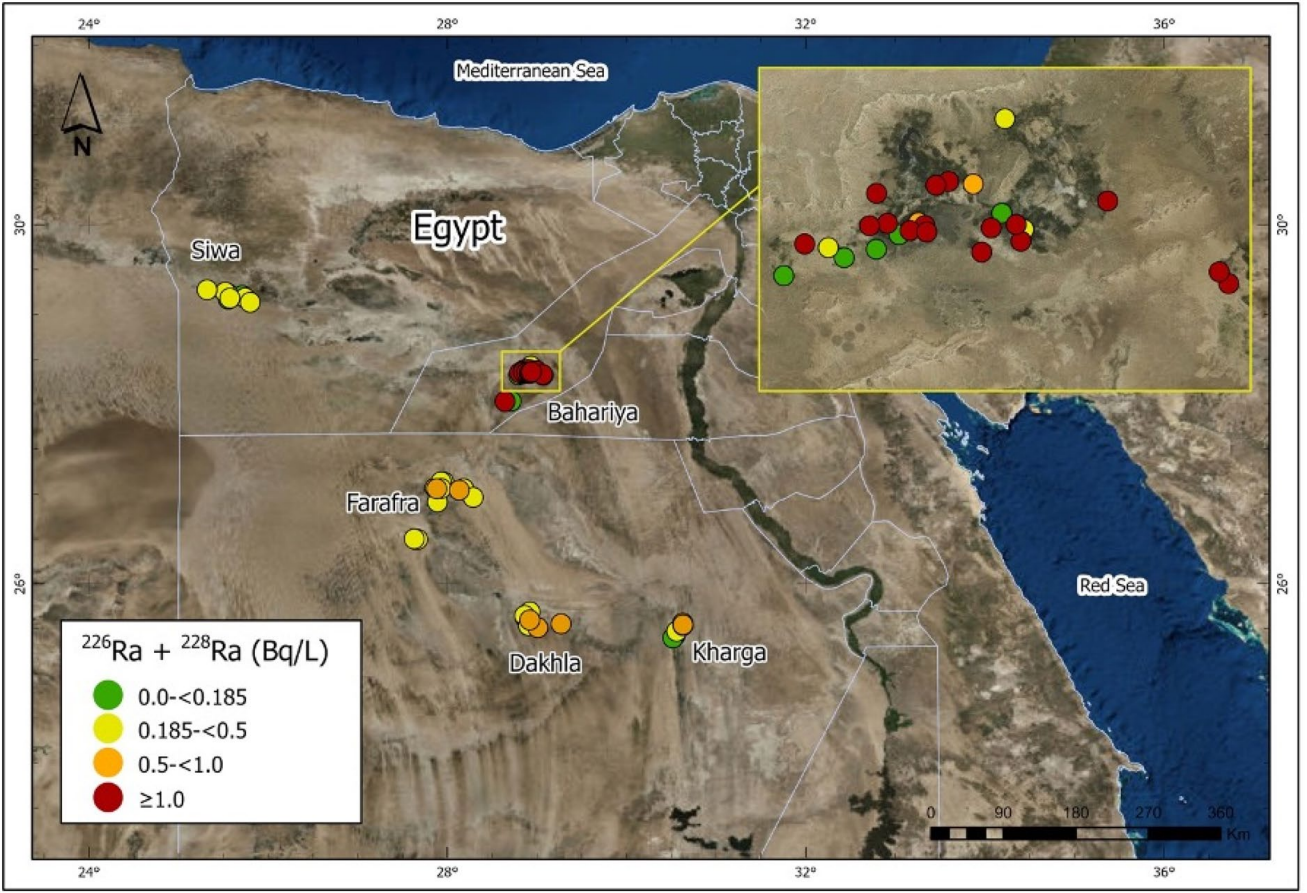
Map of Egypt, showing the locations of the investigated groundwater wells in the Western Desert. Activities of ^226^Ra + ^228^Ra are indicated by colored symbols. (mapped by using ArcGIS Pro 2.6.2; URL: https://pro.arcgis.com/en/pro-app/get-started/get-started.htm).

**Figure 4:**
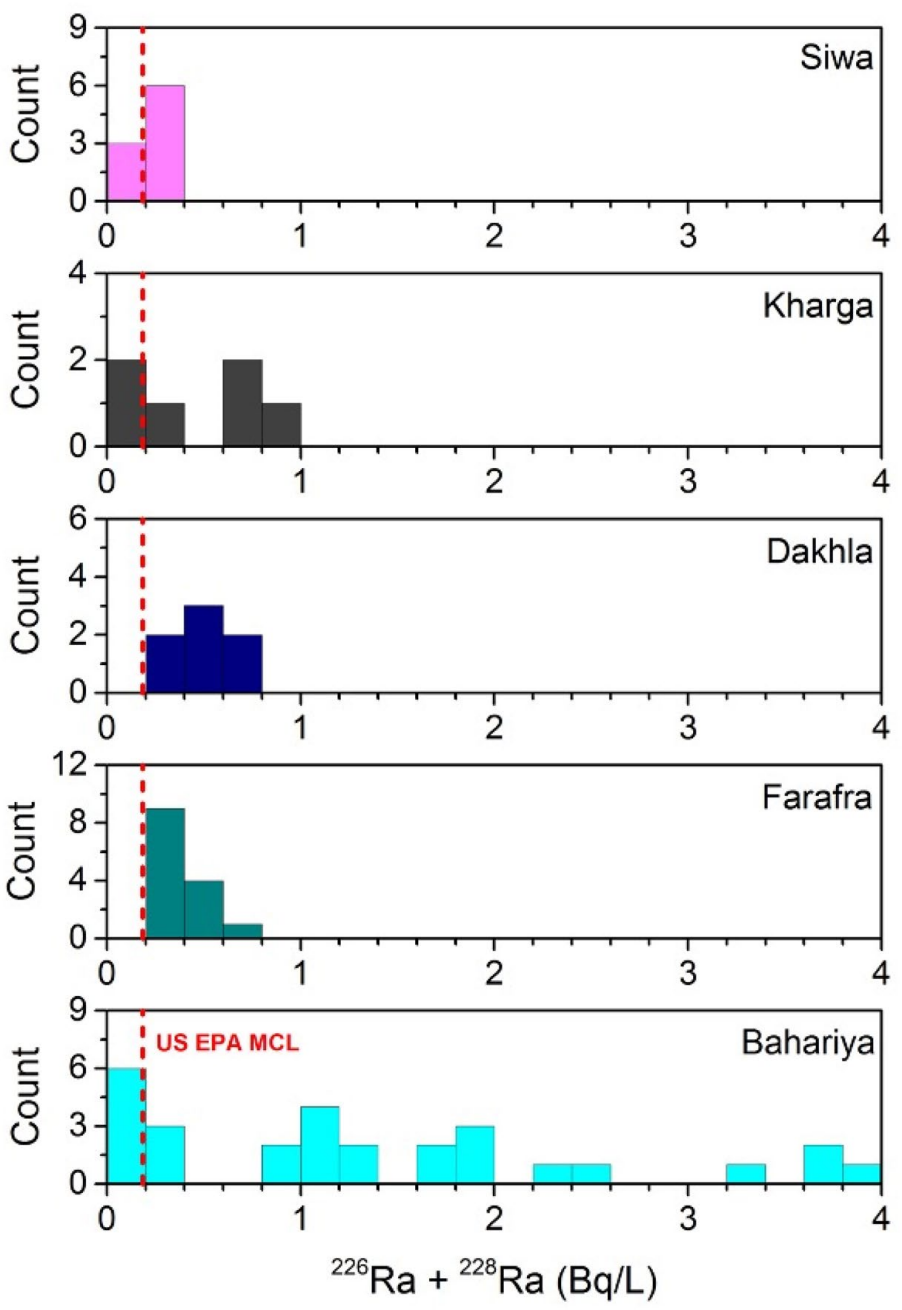
Histograms showing frequencies of total Ra activities in groundwater from the Western Desert of Egypt. The majority of wells have values exceeding the drinking water MCL of the US EPA (0.185 Bq/L for combined ^226^Ra + ^228^Ra; vertical dashed line). The total Ra activities in some Bahariya wells are > 20 times higher than this MCL value.

**Figure 5:**
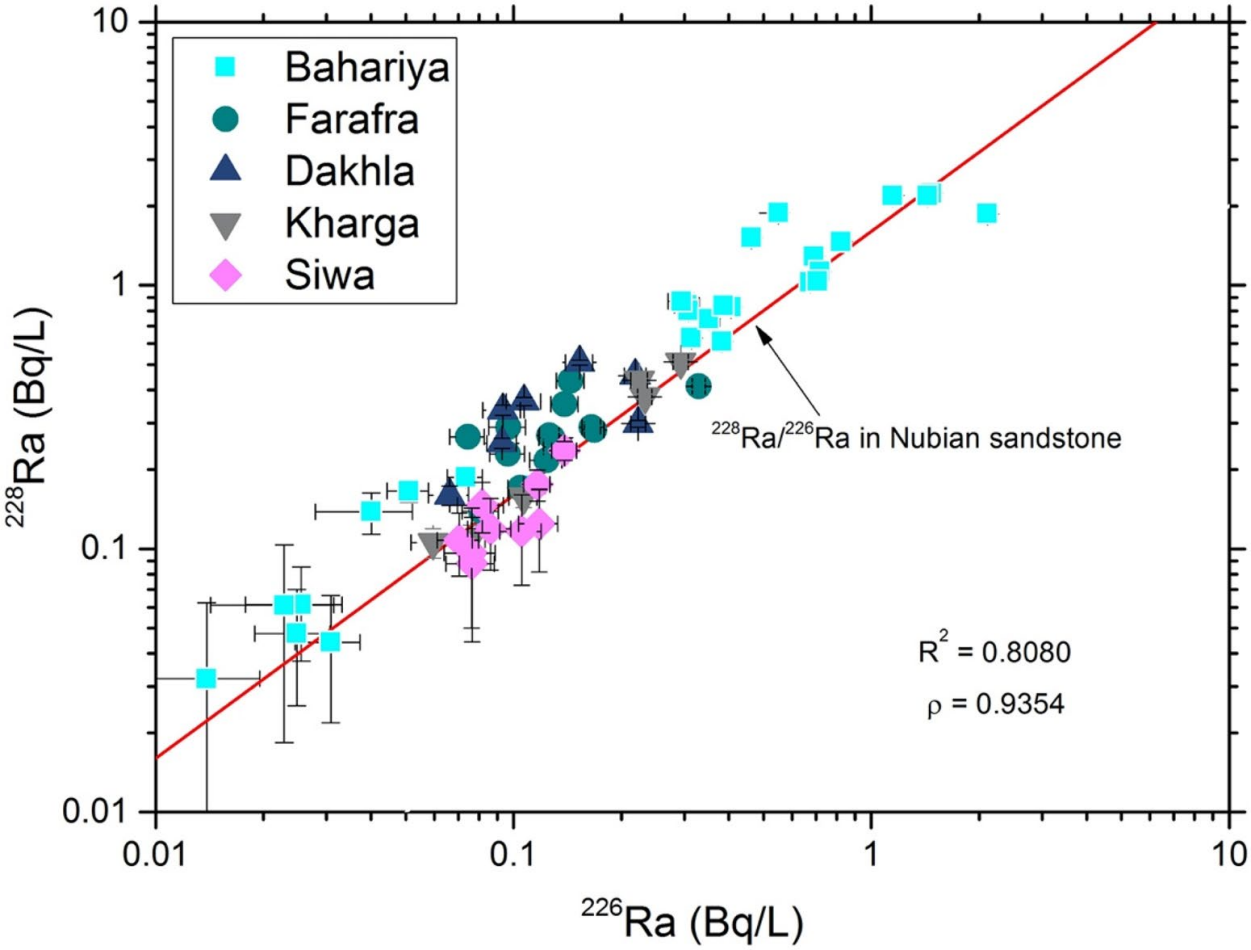
^228^Ra activity (Bq/L) versus ^226^Ra activity (Bq/L) in Western Desert groundwater samples. Reference value for ^228^Ra/^226^Ra ratio of Nubian Aquifer sandstone is from Vengosh et al. (2009). Values of *R*^2^ and ρ refer to Pearson linear and Spearman rank correlation coefficients, respectively, in this plot and following plots.

Radium isotope data for 64 groundwater samples from wells tapping the Nubian Aquifer in the Western Desert of Egypt (Fig. 3) are presented in Table S1 and summarized in Figs. 4 and 5. Activities of ^226^Ra range from 0.01 to 2.11 Bq/L and activities of ^228^Ra range from 0.03 to 2.31 Bq/L. The highest values were measured in samples from Bahariya, and most of those from Siwa had relatively low Ra activities compared to the other locations (Fig. 4). The majority of samples had Ra activities exceeding the maximum contaminant levels (MCL) for drinking water of the US Environmental Protection Agency, the European Union, and the World Health Organization (Table S1 and Fig. 4). The highest activities for ^226^Ra + ^228^Ra were in excess of 20 times the MCL at some locations (Table S1, Fig. 4). Elevated activities of Ra observed in the Western Desert (this study) are consistent with other reported Ra data for groundwaters from the Nubian Aquifer in the Eastern Desert^8^ and Sinai Peninsula of Egypt^7^ and elsewhere in the Middle East, i.e. in Negev Desert of Israel^4,5^, Disi Aquifer of Jordan^3^, and Saq Aquifer of northern Saudi Arabia^6^. On a regional scale, Ra activities in old groundwaters of the Middle East and northeast Africa generally exceed those reported for other sandstone aquifers worldwide (Table S2)**.** The relatively narrow range of ^228^Ra/^226^Ra activity ratios of groundwaters from the investigated areas along with other published data from the Middle East implies similarity in geology and lithology of the Nubian Aquifer rocks and perhaps convergence of geochemical conditions for these aquifers across the MENA region.

The distribution and behavior of Ra isotopes in groundwaters is controlled by the coupled effects of multiple geochemical processes. Radium isotopes are introduced to groundwater by α-recoil upon decay of thorium parent nuclides in the aquifer solids, by desorption of Ra from surfaces of clays and oxides in the aquifer materials, and by dissolution of Ra-bearing mineral phases. At steady state, the rate of Ra input to water is balanced by its radioactive decay and removal by sorption to or coprecipitation with aquifer solids. Sorption capacity of aquifer solids for Ra is generally a function of the abundance of clays and oxyhydroxides, as well as pH, temperature, redox potential, and salinity^15,16^. Clay minerals can scavenge Ra by adsorption^17^ whereas hydrous Fe- and Mn-oxides can control Ra release and uptake through pH-dependent desorption and adsorption, respectively^18–20^. Radium is strongly adsorbed to aquifer solids in low-salinity, near-neutral pH, oxic groundwater^3,16,21–26^. Groundwater salinity promotes Ra desorption due to the competition of the more abundant cations for sorption sites^2,15,27,28^. Reductive dissolution of Fe- and Mn-oxides, which usually hold more Ra than the surrounding rock matrix^29^, releases Ra to the water^30^. Further, this process removes a major sorbent from the aquifer matrix that might otherwise potentially limit Ra in solution that is released by α-recoil. Co-precipitation with sulfate minerals may also exert a significant control on dissolved Ra activity^31–33^.

Radium isotope ratios are used to decipher sources of Ra and water–rock interactions within an aquifer. The ^228^Ra/^226^Ra ratio in silicate aquifers derived from continental rocks tends to be relatively high because of their relatively high Th/U ratios^3,34^, but these ratios are relatively low in carbonate aquifers^35,36^. Groundwater samples from this study have ^228^Ra/^226^Ra activity ratios ranging from 0.89 to 3.60 (Table S1 and Fig. 5). These values are clustered around the mean value of ^228^Ra/^226^Ra activity ratios reported for rocks of the Lower Cretaceous Nubian sandstone aquifer in Negev, Israel and the Disi sandstone aquifer in Jordan (~ 1.6;^3^). This may indicate a state of radioactive equilibrium between daughter products (^228^Ra and ^226^Ra) and their parent nuclides (^232^Th and ^238^U) in the aquifer solids. These ^228^Ra/^226^Ra ratios are consistent with the relatively high Th/U ratio in the aquifer solids, which are derived from Proterozoic crystalline rocks.

**Figure 6:**
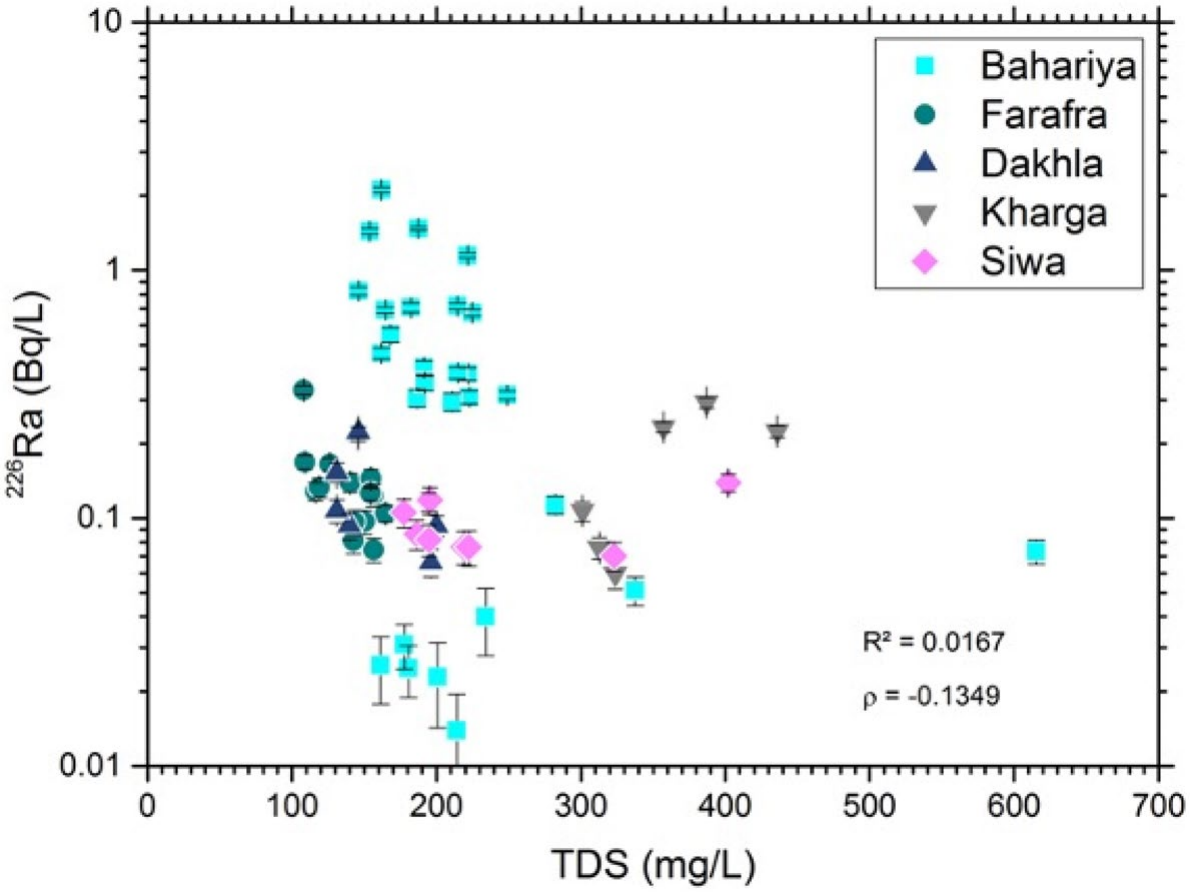
Diagram showing the relationship between ^226^Ra activities (Bq/L) and total dissolved solids (mg/L) in Western Desert groundwaters.

Groundwater samples from the Nubian Aquifer have generally low concentrations of total dissolved solids (TDS), ranging from 108 to 615 mg/L (Table S3). The data for Ra activities and TDS show no apparent correlation (Fig. 6). This may imply a low abundance of clays and Fe–Mn oxide minerals that provide sorption sites for Ra. It is also possible that Ra activity could be controlled partly by cation exchange reactions between the surfaces of clay minerals, iron oxyhydroxides, and organic substances and dissolved aqueous species^37–39^. These reactions involve reversible exchange of adsorbed ionic species resulting in compositional changes of both phases. For instance, divalent alkaline earth cations (i.e., Ca^2+^ and Mg^2+^) in solution tend to adsorb to negatively-charged clay mineral surfaces, displacing monovalent alkali cations (i.e., Na^+^ and K^+^) into solution^40,41^. Groundwaters experiencing cation exchange reactions are typically observed to have Na/(Ca + Mg) molar ratios greater than 15^38,39^ and Na/Cl ratios higher than that of seawater (0.86) or halite (1.0)^41^. The majority of the investigated Nubian Aquifer wells do not show substantial variations in either Na/(Ca + Mg) or Na/Cl molar ratios. The median values for these ratios are 1.1 and 0.3, 1.0 and 0.7, 0.7 and 0.57, 1.4 and 1.15, and 1.7 and 0.5 for Bahariya, Farafra, Dakhla, Kharga, and Siwa respectively (Table S3). These results indicate that cation exchange with clay minerals is not a significant control of solute composition in this aquifer system. This is consistent with the nearly monomineralic composition of the Nubian aquifer sandstone that is composed mainly of quartz with only a few thin clay-rich interlayers^42^.

**Figure 7:**
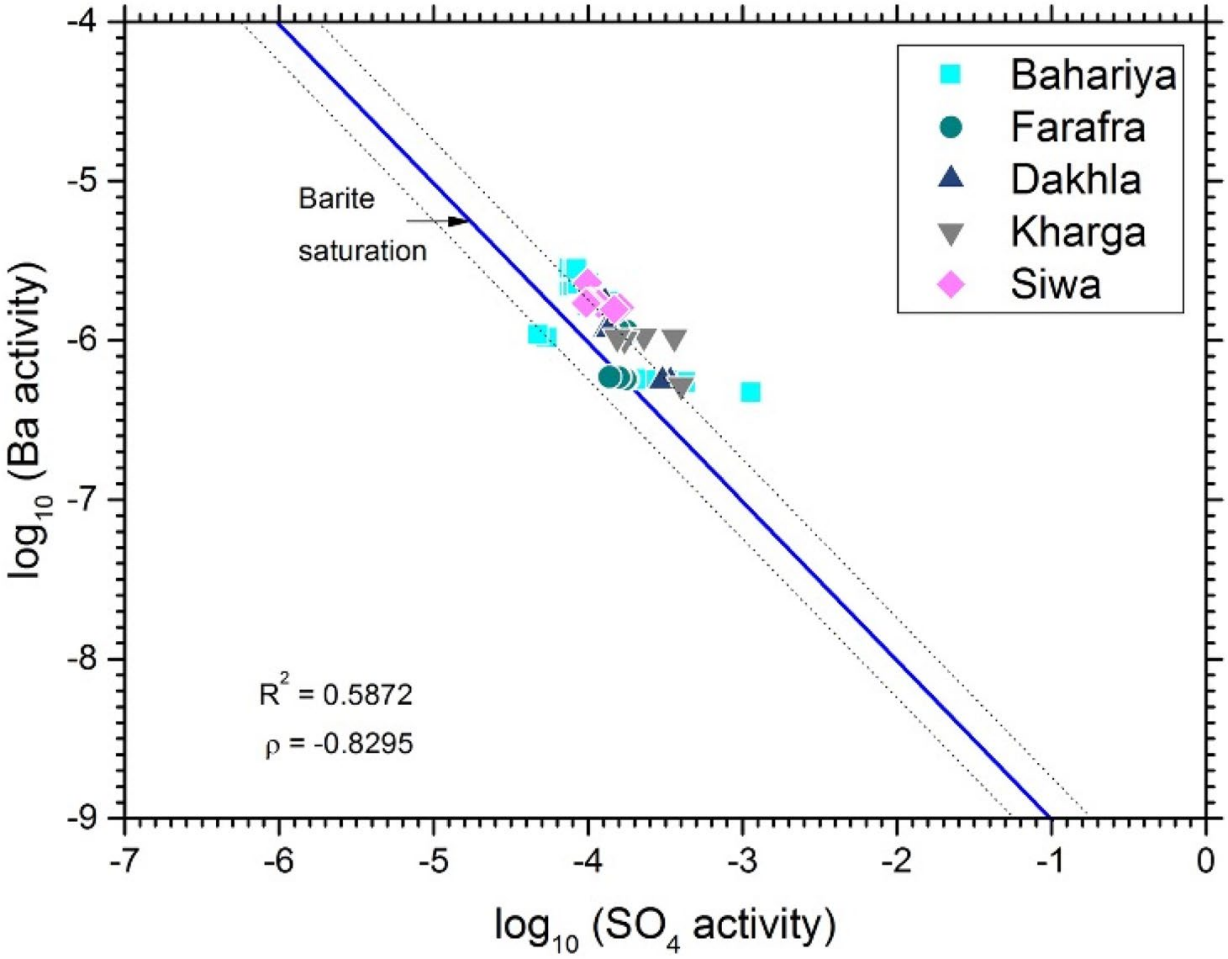
Relationship between log activities of SO_4_ and Ba in Western Desert groundwater samples. All groundwater samples are near saturation with barite. Dashed lines are (± 0.25) error bands.

**Figure 8:**
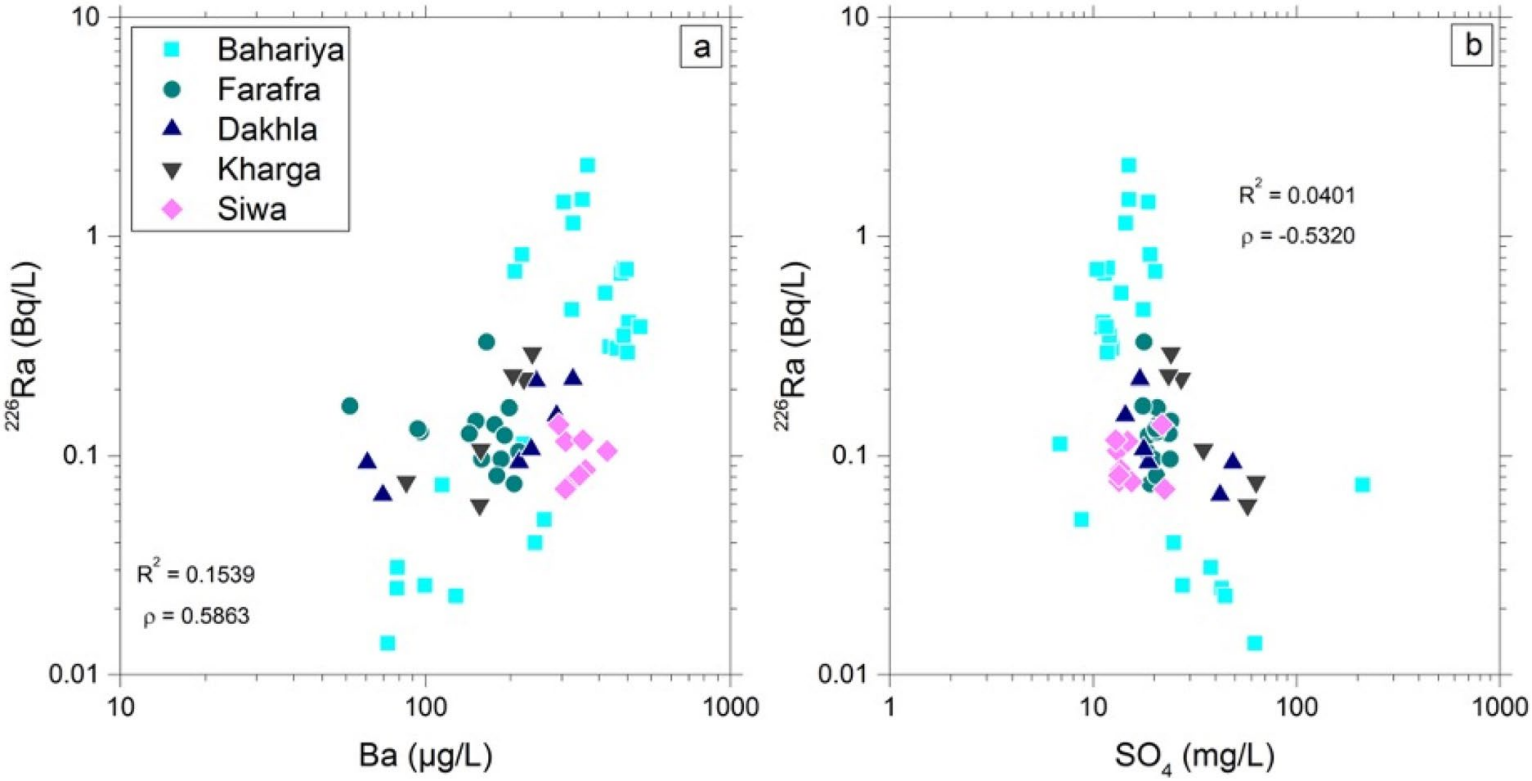
Relationships between a) ^226^Ra (Bq/L) and Ba (μg/L), and b) ^226^Ra (Bq/ L) and SO_4_ (mg/L) in Western Desert groundwater samples.

Radium substitutes for Ba in the barite crystal structure, so precipitation and dissolution of barite may control the aqueous concentration of Ra^3,7,8,26,33,43–45^. The relationship between thermodynamic activities of Ba and SO_4_ in Nubian Aquifer groundwaters indicates that they are approximately saturated with barite (Fig. 7). Saturation indices are <  < 1 for other potential Ra host minerals such as calcite, gypsum, anhydrite, and celestine (Table S4). In addition, the positive correlation between Ra and Ba (Fig. 8a) and the negative correlation between Ra and SO_4_ (Fig. 8b), although weaker than that between Ba and SO_4_ (Fig. 7), are both consistent with control of dissolved Ra activity by barite precipitation/dissolution. There are several indicators of anoxic conditions in the aquifer that could drive reduction of SO_4_ leading to destabilization of barite and release of Ra to water. The elevated Fe and Mn concentrations and low U concentrations (Table S1) are particularly diagnostic of anoxic conditions. Continual precipitation/dissolution of barite near equilibrium would act to buffer dissolved Ra activity, with local variations dependent on the flux of Ra isotopes into the water from alpha-recoil and dissolution of trace minerals containing elevated concentrations of U- and Th-series radionuclides. The highest Ra isotope activities were measured in the anoxic groundwater where Fe concentrations indicate Fe-reducing conditions and SO_4_ concentrations are relatively low (Fig. S1 in Supplementary Material). Anoxic conditions typically develop in confined aquifers^33,39,46^ and in deeper parts of unconfined portions of an aquifer system^47^. Reducing conditions in confined aquifers are commonly promoted by anaerobic bacterial metabolism, whereby different strains of bacteria use Fe–Mn oxyhydroxides and SO_4_ as terminal electron acceptors. The reduction of Fe–Mn minerals and sulfate minerals within the aquifer solids acts to increase Ra activity in the aquifer water.

Vertical and lateral heterogeneities in the Nubian Aquifer lithologies are likely to affect the local Ra isotope activities over length scales commensurate with those of the heterogeneous features. An important factor that could affect the local ^228^Ra/^226^Ra ratio of groundwater is the decay constant of each isotope relative to the groundwater flow velocity. Differences in mineralogy, grain size and porosity distribution, pH, redox conditions may all affect the input and removal of Ra in groundwater. However, detailed investigations of the lithologic heterogeneities in the Nubian Aquifer were not included in the present study.

Comparisons of the Ra activities with TDS and sulfate concentrations in groundwater from the Nubian Aquifer and correlative aquifer formations in Egypt, Jordan, and Saudi Arabia are shown in Figs. S2, S3, and S4, using data from^3,6–8^. The strong correlation between ^226^Ra and ^228^Ra activity was evident throughout the region, although with somewhat weaker correlation reported for the Saq Aquifer in northern Saudi Arabia (Fig. S2 in Supplementary Material). The range in ^226^Ra activities was similar in all locations from ~ 0.01 to ~ 1 Bq/L, with several samples having activities > 1 Bq/L in Jordan and the Western Desert of Egypt, and there is no overall correlation between ^226^Ra and TDS despite a nearly two order of magnitude range in TDS from near 100 mg/L in some of the Western Desert samples to near 10,000 mg/L in one of the Sinai samples (Fig. S3 in Supplementary Material). The weak negative correlation of Ra with sulfate in the Western Desert samples is not present in the samples from other locations (Fig. S4 in Supplementary Material). The strong correlation of Ba and Ra observed in the Western Desert samples is not present in the other locations, and Ba concentrations in the Western Desert samples are roughly 10 times greater than those of the other locations (Fig. S5 in Supplementary Material).

### Annual radiation dose rates from ingestion of untreated Nubian Aquifer water

The Western Desert occupies an area of 700,000 km^2^, comprising two-thirds of the total land of Egypt. Population density of the Western Desert is concentrated in oasis areas with a range of 5–250 people/km^2^^48^. Population centers of the Western Desert depend entirely on fossil groundwater from the Nubian Aquifer for domestic and agricultural purposes. The World Health Organization (WHO) provides guidance on radiation dose from drinking water to minimize health risks of radiation exposure. An individual dose criterion (IDC) of 0.1 mSv/yr is regulated, representing a level of risk not expected to cause any observable adverse health impacts^49^.

**Figure 9:**
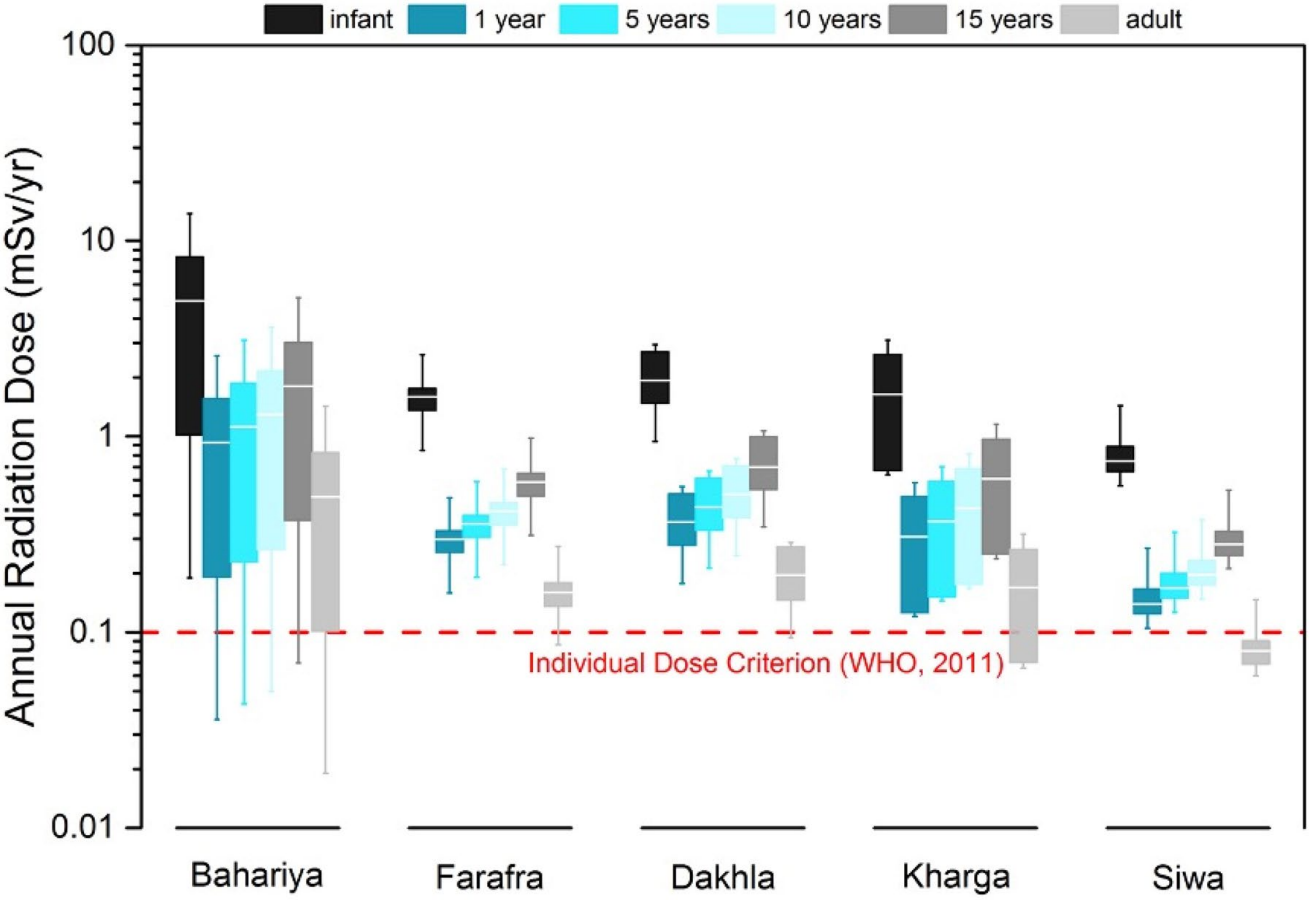
Annual radiation dose rates from ingestion of untreated Nubian Aquifer groundwater in the studied population centers for different life ages of population including infants (< 1 year), children (1–15 years), and adults (> 15 years). Doses are calculated based on the dose coefficients by the International Commission on Radiological Protection to members of the public^50^. Conservative water consumption rates of 2 L/day for adults, 1 L/day for children, and 0.5 L/day for infants are assumed. Dashed line indicates the individual dose criterion (IDC) at 0.1 mSv/yr^49^.

Annual effective doses from utilization of untreated Nubian Aquifer groundwater in the Western Desert have been estimated for different life ages of population, including infants (< 1 year), children (1–15 years), and adults (> 15 years), as shown in Fig. 9 and Table S5. These values are calculated based on the reported dose coefficients by the International Commission on Radiological Protection (ICRP) to members of the public^50^, assuming conservative water consumption rates of 0.5 L/day for infants, 1.0 L/day for children, and 2.0 L/day for adults. The annual radiation dose estimates for infants and adults at the Bahariya oasis range from 1.9 to 137.7 and 0.2 to 14.3 times the WHO’s guidance levels^49^, respectively (Table S5). In Farafra, radiation doses range from 8.5 to 26.1 and 0.9 to 2.8 times the recommended level for infants and adults, respectively (Table S5). In Dakhla, radiation doses for infants and adults vary from 9.4 to 29.4 and 0.9 to 2.9 times the recommended level, respectively (Table S5). The annual radiation dose in Kharga ranges from 6.4 to 31.1 and 0.7 to 3.2 times the recommended level for infants and adults, respectively (Table S5). The radiation dose in Siwa is the lowest among the investigated locations with an annual dose varies from 5.6 to 14.4 and 0.6 to 1.5 times the guidance level for infants and adults, respectively (Table S5). In all investigated sites the annual dose estimates for infants exceed the WHO guidelines. The estimates provided here account only for the dose received through drinking water, and thereby may be biased low as the dose from food intake is not included. Further investigation of radium uptake by soil and transfer to vegetables during irrigation is still needed for more accurate radiation dose estimates.

The elevated natural radioactivity indicates that groundwater from the Nubian Aquifer in the Western Desert of Egypt should be used with caution for domestic and agricultural purposes, and Ra removal may be necessary before water is used for human consumption. This study has detected several locations where Ra activities are below the MCL for drinking water (Fig. 4). Blending of low-Ra water with high-Ra water, where this is possible, may be the most cost-effective method for reducing Ra activities below regulatory limits for drinking water. This approach will require monitoring of Ra activities in all groundwater wells used for domestic and agricultural production in the Western Desert. Alternative methods for Ra mitigation could include precipitation of Fe- and Mn-oxides from produced groundwater by aeration followed by Ra adsorption and removal by settling or filtration of the precipitates, or removal of Ra by other treatments such as ion exchange or reverse osmosis at the point of use. Findings from this study indicate that monitoring and mitigation of natural radioactivity are essential components of water quality management for global groundwater reserves, which consist primarily of fossil groundwaters^51^.

## Methods

Fieldwork was conducted in January 2016 and August 2017 to sample groundwater from deep drilled wells tapping the Nubian Aquifer in the Western Desert of Egypt. A total of 64 groundwater locations were sampled for radium isotopes and bulk chemistry analyses from the Bahariya, Farafra, Dakhla, Kharga, and Siwa areas. Sampled groundwater wells had total depth (TD) ranging between 800 and 1500 m and depth to the water level (DWL) between 500 and 800 m.

For analysis of ^226^Ra and ^228^Ra, 25 L of water was collected from each groundwater well and poured into a large open container prior to extraction of radium by adsorption on Mn-oxide coated acrylic fiber^18,52,53^. Water was aerated during filling of the container to cause degassing and oxidation to help ensure quantitative extraction of Ra by the Mn-fiber as recommended^53^. Turbidity was negligible and water was not filtered. The water was passed slowly (< 1 L per minute) by gravity feed through ¼-inch plastic tubing into the inlet of a 100 cm^3^ flow-through cartridge containing 14 g of fluffed Mn-coated acrylic fiber (Scientific Computer Instruments, Columbia, SC). The Mn-fiber adsorbed Ra from the water. After draining and removal from the cartridge, the Mn-fiber was transferred to a labeled plastic zip-loc bag for transport to the laboratory. Extraction efficiency was evaluated in this study by connecting two Mn-fiber sampling cartridges in series, then measuring each separately after processing a water sample through both cartridges. The upstream cartridge retained all of the Ra, and the downstream cartridge had no detectable Ra, indicating that our normal sampling method using a single Mn-fiber cartridge yielded essentially quantitative extraction from the water sample. A similar sampling procedure has been used extensively for seawater and groundwater by others and found to be quantitative when flow rate through the Mn-fiber column is maintained at < 1–2 L per minute^52,53^

Measurement of Ra isotopes by gamma spectrometry followed the same procedure used previously in our laboratory^8^. The Mn-fiber samples were sealed in labeled aluminum containers and measured by gamma spectrometry using a low-background Canberra model CR-3020 reverse-electrode HPGe detector enclosed in a 10-cm thick Pb shield. Detector output signal was connected to an EG&G Ortec DSPEC-LF digital gamma spectrometer interfaced to a PC for spectral analysis using Maestro multichannel analyzer software. Data were acquired for at least 18 h. ^226^Ra was measured from its gamma emission at 186.2 keV, and ^228^Ra was measured from its ^228^Ac daughter gamma emission at 911.3 keV. Activity of ^234^Th at 63.3 keV was undetectable in all samples, indicating negligible adsorption of U on the Mn fiber, obviating an interference correction for the ^235^U photopeak at 185.7 keV. Measurements of ^226^Ra and ^228^Ra were calibrated with certified NIST-4965 and NIST-4339b Standard Radium Solutions (US National Institute of Standards and Technology) adsorbed on 14 g of Mn-fiber sealed in an identical aluminum container and counted in the same geometry as the samples. Standard activities were corrected for decay between time of certification and time of measurement. Measurement of a Mn-fiber blank showed ^226^Ra and ^228^Ra activities indistinguishable from detector background. Reported activities were corrected for detector background and decay from time of sample collection to time of analysis. Reported errors are one standard deviation from counting statistics. Detection limits were 5 mBq/L for ^226^Ra and 2 mBq/L for ^228^Ra. The activities of ^226^Ra and ^228^Ra in replicate samples agreed within their respective 95% confidence limits.

Chemical analysis of water samples followed methods used previously by our laboratory^8^. Samples were collected in 50-mL centrifuge tubes, one filtered and acidified using ultrapure nitric acid and one unfiltered. Cations were measured in the filtered, acidified samples by using inductively coupled plasma mass spectrometry and microwave plasma atomic emission spectroscopy. Anions were measured in the unfiltered samples by using ion chromatography. Accuracies of cation and anion analyses ranged from ± 2–10%. Temperature and pH were measured in the field. Total alkalinity expressed as bicarbonate was measured by titration with 0.1 M HCl using NIST-traceable pH calibration solutions.

Mineral-saturation indices (SI) were calculated by using the solubility and aqueous speciation modeling capabilities of PHREEQC version 3 code^54^.

## Supplementary Information

Supplementary Information.
