# Supplementary material for: Elevated radium levels in Nubian Aquifer groundwater of Northeastern Africa
Source: Sci Rep. 2021 Jan 8;11:78. doi: 10.1038/s41598-020-80160-0 (PMC7794551; doi:10.1038/s41598-020-80160-0)
Supplement: Supplementary file 1 — Supplementary Information. [file 41598_2020_80160_MOESM1_ESM.docx]

**SUPPLEMENTARY INFORMATION**

**Elevated Radium Levels in Nubian Aquifer Groundwater of Northeastern Africa**

Mahmoud I. Sherif^1,2,^* and Neil C. Sturchio^1^

1. Dept. of Earth Sciences, University of Delaware, Newark, DE 19716, USA

2. Dept. of Geology, Tanta University, Tanta 31527, Egypt

*Corresponding author at: Department of Earth Sciences, University of Delaware, Newark, DE 19716, USA. E-mail address: [sherif@udel.edu](mailto:sherif@udel.edu) (Mahmoud I. Sherif)

**Figure S1. Relationship between concentrations of Fe (µg/L) and SO_4_ (mg/L) for groundwater samples. Solid symbols, ^226^Ra + ^228^Ra activities > 0.185 Bq/L; open symbols, ^226^Ra + ^228^Ra activities < 0.185 Bq/L. Samples having ^226^Ra + ^228^Ra activities > 0.185 Bq/L are mostly from groundwater wells having reducing conditions, whereas those having ^226^Ra + ^228^Ra activities < 0.185 Bq/L are mostly oxic. The dividing line is based on [1].**

 **Figure S2. ^228^Ra activity (Bq/L) versus ^226^Ra activity (Bq/L) in groundwater wells from this study in comparison with compiled data of correlative fossil groundwaters across the region. Data for other localities are from referenced [3–6]. Reference Nubian sandstone ratio of ^228^Ra/^226^Ra = 1.6 is from reference [4].**

**Figure S3. ^228^Ra activity (Bq/L) versus TDS (mg/L) in groundwater samples from this study in comparison with compiled data of correlative fossil groundwaters across the region. Data for other localities are from references [3–6].**

**Figure S4. ^228^Ra activity (Bq/L) versus SO_4_ (mg/L) in groundwater samples from this study in comparison with compiled data of correlative fossil groundwaters across the region. Data for other localities are from references [3–5].**

**Figure S5. ^228^Ra activity (Bq/L) versus Ba (μg/L) in groundwater samples from this study in comparison with compiled data of correlative fossil groundwaters across the region. Data for other localities are from references [3–5].**

Table**S1. Radium isotope data for groundwater from the Western Desert, Egypt.**

| **Sample ID** | **^226^Ra (Bq/L)** | **^228^Ra (Bq/L)** | **^228^Ra/^226^Ra**  **(activity ratio)** | **%EPA^a^** | **%EU^b^** | **%WHO^c^** |
| --- | --- | --- | --- | --- | --- | --- |
| NS1-Bahariya | 0.05 ± 0.01 | 0.17 ± 0.02 | 3.24 | 117 | 93 | 171 |
| NS2-Bahariya | 0.31 ± 0.01 | 0.63 ± 0.01 | 2.01 | 510 | 378 | 662 |
| NS3-Bahariya | 0.01 ± 0.01 | 0.03 ± 0.03 | 2.31 | 25 | 19 | 33 |
| NS4-Bahariya | 0.02 ± 0.01 | 0.05 ± 0.02 | 1.92 | 39 | 29 | 50 |
| NS5-Bahariya | 1.47 ± 0.03 | 2.24 ± 0.01 | 1.52 | 2004 | 1412 | 2382 |
| NS6-Bahariya | 0.69 ± 0.02 | 1.29 ± 0.01 | 1.86 | 1070 | 783 | 1358 |
| NS7-Bahariya | 0.68 ± 0.02 | 1.03 ± 0.01 | 1.52 | 922 | 650 | 1097 |
| NS8-Bahariya | 0.72 ± 0.02 | 1.13 ± 0.01 | 1.57 | 998 | 707 | 1199 |
| NS9-Bahariya | 1.15 ± 0.03 | 2.19 ± 0.01 | 1.91 | 1804 | 1325 | 2305 |
| NS10-Bahariya | 0.03 ± 0.01 | 0.04 ± 0.02 | 1.43 | 41 | 28 | 47 |
| NS11-Bahariya | 0.03 ± 0.01 | 0.06 ± 0.02 | 2.41 | 47 | 36 | 64 |
| NS12-Bahariya | 0.46 ± 0.02 | 1.52 ± 0.01 | 3.28 | 1069 | 850 | 1562 |
| NS13-Bahariya | 0.38 ± 0.02 | 0.61 ± 0.01 | 1.60 | 538 | 383 | 651 |
| NS14-Bahariya | 0.41 ± 0.02 | 0.83 ± 0.02 | 2.04 | 666 | 494 | 866 |

Table S1 continued

| **Sample ID** | **^226^Ra (Bq/L)** | **^228^Ra (Bq/L)** | **^228^Ra/^226^Ra**  **(activity ratio)** | **%EPA^a^** | **%EU^b^** | **%WHO^c^** |
| --- | --- | --- | --- | --- | --- | --- |
| NS15-Bahariya | 0.31 ± 0.02 | 0.84 ± 0.01 | 2.75 | 619 | 481 | 871 |
| NS16-Bahariya | 0.02 ± 0.01 | 0.06 ± 0.04 | 2.67 | 45 | 35 | 63 |
| NS17-Bahariya | 2.11 ± 0.05 | 1.87 ± 0.01 | 0.89 | 2152 | 1357 | 2081 |
| NS18-Bahariya | 0.07 ± 0.01 | 0.19 ± 0.01 | 2.54 | 141 | 108 | 194 |
| NS19-Bahariya | 0.31 ± 0.02 | 0.80 ± 0.01 | 2.61 | 600 | 463 | 834 |
| NS20-Bahariya | 0.82 ± 0.03 | 1.47 ± 0.01 | 1.78 | 1238 | 898 | 1548 |
| NS21-Bahariya | 1.44 ± 0.04 | 2.19 ± 0.01 | 1.53 | 1962 | 1384 | 2337 |
| NS22-Bahariya | 0.71 ± 0.03 | 1.04 ± 0.01 | 1.47 | 943 | 660 | 1107 |
| NS23-Bahariya | 0.35 ± 0.02 | 0.75 ± 0.02 | 2.12 | 593 | 443 | 781 |
| NS24-Bahariya | 0.39 ± 0.02 | 0.84 ± 0.02 | 2.18 | 662 | 497 | 878 |
| NS25-Bahariya | 0.55 ± 0.04 | 1.88 ± 0.02 | 3.42 | 1316 | 1052 | 1940 |
| NS26-Bahariya | 0.04 ± 0.01 | 0.14 ± 0.02 | 3.46 | 96 | 77 | 142 |
| NS27-Bahariya | 0.11 ± 0.01 | 0.17 ± 0.01 | 1.54 | 155 | 110 | 185 |
| NS28-Bahariya | 0.29± 0.02 | 0.87 ± 0.02 | 2.95 | 629 | 494 | 899 |

Table S1 continued

| **Sample ID** | **^226^Ra (Bq/L)** | **^228^Ra (Bq/L)** | **^228^Ra/^226^Ra**  **(activity ratio)** | **%EPA^a^** | **%EU^b^** | **%WHO^c^** |
| --- | --- | --- | --- | --- | --- | --- |
| NS29-Farafra | 0.14 ± 0.01 | 0.35 ± 0.01 | 2.55 | 266 | 205 | 368 |
| NS30-Farafra | 0.33 ± 0.01 | 0.41 ± 0.01 | 1.26 | 402 | 273 | 446 |
| NS31-Farafra | 0.10 ± 0.01 | 0.17 ± 0.01 | 1.63 | 148 | 106 | 180 |
| NS32-Farafra | 0.12 ± 0.01 | 0.22 ± 0.01 | 1.75 | 184 | 133 | 229 |
| NS33-Farafra | 0.07 ± 0.01 | 0.27 ± 0.01 | 3.57 | 184 | 148 | 273 |
| NS34-Farafra | 0.10 ± 0.01 | 0.23 ± 0.01 | 2.38 | 176 | 134 | 239 |
| NS35-Farafra | 0.08 ± 0.01 | 0.14 ± 0.02 | 1.71 | 119 | 86 | 147 |
| NS36-Farafra | 0.14 ± 0.01 | 0.43 ± 0.01 | 3.01 | 313 | 246 | 449 |
| NS37-Farafra | 0.10 ± 0.01 | 0.29 ± 0.01 | 2.99 | 208 | 164 | 298 |
| NS38-Farafra | 0.13 ± 0.01 | 0.27 ± 0.01 | 2.13 | 213 | 160 | 281 |
| NS39-Farafra | 0.17 ± 0.01 | 0.29 ± 0.01 | 1.75 | 246 | 178 | 306 |
| NS40-Farafra | 0.13 ± 0.01 | 0.26 ± 0.01 | 2.05 | 212 | 157 | 276 |
| NS41-Farafra | 0.13 ± 0.01 | 0.24 ± 0.01 | 1.83 | 203 | 148 | 257 |
| NS42-Farafra | 0.17 ± 0.01 | 0.28 ± 0.01 | 1.67 | 243 | 175 | 299 |

Table S1 continued

| **Sample ID** | **^226^Ra (Bq/L)** | **^228^Ra (Bq/L)** | **^228^Ra/^226^Ra**  **(activity ratio)** | **%EPA^a^** | **%EU^b^** | **%WHO^c^** |
| --- | --- | --- | --- | --- | --- | --- |
| NS43-Dakhla | 0.09 ± 0.01 | 0.25 ± 0.01 | 2.71 | 186 | 144 | 261 |
| NS44-Dakhla | 0.22 ± 0.02 | 0.45 ± 0.01 | 2.08 | 364 | 271 | 476 |
| NS45-Dakhla | 0.07 ± 0.01 | 0.16 ± 0.01 | 2.41 | 122 | 93 | 166 |
| NS46-Dakhla | 0.09 ± 0.01 | 0.34 ± 0.01 | 3.60 | 232 | 186 | 345 |
| NS47-Dakhla | 0.11 ± 0.01 | 0.36 ± 0.01 | 3.38 | 254 | 203 | 373 |
| NS48-Dakhla | 0.15 ± 0.01 | 0.51 ± 0.01 | 3.33 | 358 | 285 | 524 |
| NS49-Dakhla | 0.22 ± 0.01 | 0.30 ± 0.01 | 1.34 | 282 | 194 | 321 |
| NS50-Kharga | 0.06 ± 0.01 | 0.11 ± 0.01 | 1.77 | 89 | 65 | 112 |
| NS51-Kharga | 0.08 ± 0.01 | 0.11 ± 0.01 | 1.43 | 100 | 69 | 116 |
| NS52-Kharga | 0.11 ± 0.01 | 0.16 ± 0.01 | 1.47 | 142 | 100 | 167 |
| NS53-Kharga | 0.29 ± 0.01 | 0.51 ± 0.01 | 1.75 | 435 | 314 | 541 |
| NS54-Kharga | 0.22 ± 0.01 | 0.44 ± 0.01 | 1.94 | 357 | 263 | 459 |
| NS55-Kharga | 0.23 ± 0.01 | 0.38 ± 0.01 | 1.62 | 330 | 235 | 401 |
| NS56-Siwa | 0.11 ± 0.01 | 0.12 ± 0.04 | 1.11 | 120 | 79 | 127 |

Table S1 continued

| **Sample ID** | **^226^Ra (Bq/L)** | **^228^Ra (Bq/L)** | **^228^Ra/^226^Ra**  **(activity ratio)** | **%EPA^a^** | **%EU^b^** | **%WHO^c^** |
| --- | --- | --- | --- | --- | --- | --- |
| NS57-Siwa | 0.09 ± 0.01 | 0.12 ± 0.04 | 1.38 | 111 | 77 | 128 |
| NS58-Siwa | 0.08 ± 0.01 | 0.09 ± 0.04 | 1.15 | 89 | 59 | 96 |
| NS59-Siwa | 0.12 ± 0.01 | 0.18 ± 0.02 | 1.51 | 158 | 111 | 187 |
| NS60-Siwa | 0.08 ± 0.01 | 0.10 ± 0.05 | 1.27 | 93 | 63 | 104 |
| NS61-Siwa | 0.07 ± 0.01 | 0.11 ± 0.03 | 1.53 | 96 | 68 | 115 |
| NS62-Siwa | 0.08 ± 0.01 | 0.15 ± 0.03 | 1.79 | 123 | 89 | 154 |
| NS63-Siwa | 0.14 ± 0.01 | 0.24 ± 0.02 | 1.70 | 202 | 146 | 250 |
| NS64-Siwa | 0.12 ± 0.01 | 0.12 ± 0.04 | 1.06 | 131 | 86 | 136 |
| ^a^ %EPA = (^226^Ra + ^228^Ra)/0.185 *100, ^b^ %EU = [(^226^Ra/0.5) + (^228^Ra/0.2)] * 100, ^c^ %WHO = [(^226^Ra + (^228^Ra/0.1)] * 100 | | | | | | |

**Table S2. Compiled data of Ra activities in sandstone aquifers.**

| **Aquifer System** | **^226^Ra (Bq/L)** | | | **^228^Ra (Bq/L)** | | | **^228^Ra/^226^Ra**  **(activity ratio)** |
| --- | --- | --- | --- | --- | --- | --- | --- |
|  | **Min** | **Max** | **Mean** | **Min** | **Max** | **Mean** | **Mean** |
| Nubian Aquifer, Western Desert* | 0.01 | 1.55 | 0.24 | 0.03 | 2.19 | 0.46 | 2.13 |
| Shallow Nubian Aquifer, Eastern Desert [2] | 0.02 | 0.75 | 0.25 | 0.02 | 1.42 | 0.44 | 2.95 |
| Deep Nubian Aquifer, Eastern Desert [2] | 0.02 | 0.05 | 0.03 | 0.01 | 0.25 | 0.09 | 2.65 |
| Nubian Aquifer, Sinai Peninsula, Egypt [3] | 0.01 | 0.80 | 0.30 | 0.01 | 1.03 | 0.37 | 1.39 |
| Confined Rum, Disi Aquifer, Jordan [4] | 0.43 | 1.00 | 0.68 | 1.16 | 3.11 | 1.99 | 2.93 |
| Unconfined Rum, Disi Aquifer, Jordan [4] | 0.10 | 1.27 | 0.53 | 0.20 | 2.11 | 0.91 | 1.90 |
| Saq Aquifer, Saudi Arabia [5] | 0.10 | 0.82 | 0.31 | 0.21 | 3.09 | 1.09 | 4.69 |
| Mt. Simon-Hinckley, Minnesota, USA [6] | 0.05 | 0.35 | 0.19 | 0.07 | 0.34 | 0.22 | 1.40 |
| Cambrian Ordovician Aquifer, Southeastern Wisconsin, USA [7] | 0.12 | 0.24 | 0.16 | 0.10 | 0.48 | 0.24 | 1.46 |
| Cambrian-Ordovician Aquifer, eastern Wisconsin, USA [8] | 0.03 | 0.16 | 0.09 | 0.03 | 0.12 | 0.05 | 0.62 |
| Kombolgie Aquifer, Australia [9] | 0.01 | 0.28 | 0.09 | 0.01 | 0.25 | 0.06 | 0.90 |
| Continental Interclaire, Algeria [10] | 0.02 | 0.10 | 0.06 | 0.01 | 0.13 | 0.05 | 0.84 |
| Continental Interclaire, Tunisia [10] | 0.02 | 0.57 | 0.18 | 0.00 | 0.60 | 0.19 | 0.97 |
| Fujian Province, China [11] | - | - | 0.01 | - | - | 0.02 | 1.52 |
| **Data from this study* | | | | | | | |

**Table S3. Compositional data for groundwater from the Nubian aquifer in the Western Desert, Egypt.**

| **Well ID** | NS1-Bahariya | NS2-Bahariya | NS3-Bahariya | NS4-Bahariya | NS5-Bahariya | NS6-Bahariya | NS7-Bahariya | NS8-Bahariya |
| --- | --- | --- | --- | --- | --- | --- | --- | --- |
| **TDS (mg/L)** | 337.6 | 248.9 | 214 | 180.7 | 187.5 | 164.6 | 224.9 | 214.5 |
| **T (°C)** | 36 | 42.7 | 32.2 | 31.9 | 42.5 | 36.3 | 40.8 | 44 |
| **pH** | 7.5 | 7.0 | 6.7 | 6.7 | 6.8 | 6.8 | 7.1 | 6.8 |
| **Ca (ppm)** | 18.6 | 13.2 | 16.6 | 13.9 | 9.8 | 8.4 | 11.7 | 11.5 |
| **Mg (ppm)** | 21.2 | 12.7 | 14.2 | 11.2 | 9.3 | 7.7 | 12.5 | 10.7 |
| **Na (ppm)** | 33.9 | 31.9 | 15.7 | 14.1 | 23.2 | 19.6 | 26.6 | 27.9 |
| **K (ppm)** | 24.6 | 18.7 | 14.3 | 12.6 | 13.7 | 11.2 | 16.9 | 15.2 |
| **F (ppm)** | 0.8 | 0.4 | 0.2 | 0.2 | 0.2 | 0.2 | 0.4 | 0.2 |
| **Cl (ppm)** | 61 | 70.6 | 37.2 | 33.7 | 49.3 | 31 | 60.3 | 69.1 |
| **Br (ppm)** | 0.3 | 0.3 | 0.2 | 0.1 | 0.2 | 0.1 | 0.3 | 0.3 |
| **SO_4_ (ppm)** | 8.8 | 12.5 | 62.7 | 42.9 | 15 | 20.3 | 11.4 | 11.8 |
| **HCO_3_ (ppm)** | 168.4 | 88.6 | 52.9 | 52 | 66.8 | 66.1 | 84.8 | 67.8 |
| **Ba (µg/L)** | 245.4 | 402.7 | 75.4 | 80.8 | 327.2 | 196.6 | 438.8 | 448.4 |
| **Sr (µg/L)** | 109.7 | 68.4 | 95.3 | 80.4 | 54.3 | 40.2 | 62.6 | 60.9 |
| **Fe (µg/L)** | 140 | 235 | 45 | 70 | 110 | 35 | 160 | 70 |
| **Mn (µg/L)** | 52.1 | 321.6 | 402.4 | 353.7 | 534.5 | 570.7 | 257.9 | 588.5 |
| **U (µg/L)** | <0.05 | <0.05 | <0.05 | <0.05 | <0.05 | <0.05 | <0.05 | <0.05 |

Table S3 continued

| **Well ID** | NS9-Bahariya | NS10-Bahariya | NS11-Bahariya | NS12-Bahariya | NS13-Bahariya | NS14-Bahariya | NS15-Bahariya | NS16-Bahariya |
| --- | --- | --- | --- | --- | --- | --- | --- | --- |
| **TDS (mg/L)** | 221.5 | 177.7 | 161.4 | 162 | 221.8 | 191.4 | 186.5 | 200.6 |
| **T (°C)** | 41.4 | 31 | 29.8 | 42.3 | 44 | 46 | 46 | 30 |
| **pH** | 7 | 6.6 | 6.9 | 6.8 | 7.4 | 7.2 | 7.1 | 7.2 |
| **Ca (ppm)** | 11.9 | 12.8 | 13.6 | 9.6 | 1.4 | 1 | 1 | 1.7 |
| **Mg (ppm)** | 11.5 | 9.7 | 8.7 | 9.1 | 12.2 | 9.3 | 9 | 13.2 |
| **Na (ppm)** | 26.1 | 13.9 | 14.2 | 16.7 | 12.9 | 11.5 | 11.3 | 6.2 |
| **K (ppm)** | 15.8 | 11.9 | 11 | 11.4 | 18.9 | 16.6 | 15.9 | 17.4 |
| **F (ppm)** | 0.4 | 0.2 | 0.2 | 0.2 | 0.3 | 0.2 | 0.2 | 0.2 |
| **Cl (ppm)** | 52.4 | 34.3 | 36.9 | 28.9 | 83.4 | 69.3 | 63.9 | 37.8 |
| **Br (ppm)** | 0.3 | 0.2 | 0.2 | 0.1 | 0.2 | 0.2 | 0.2 | <0.1 |
| **SO_4_ (ppm)** | 14.5 | 37.9 | 27.6 | 17.7 | 11 | 11.2 | 11.9 | 44.7 |
| **HCO_3_ (ppm)** | 88.6 | 56.8 | 49 | 68.3 | 81.5 | 72 | 73.2 | 79.5 |
| **Ba (µg/L)** | 305.4 | 81 | 99.7 | 302 | 492.8 | 464.5 | 443.6 | 125.9 |
| **Sr (µg/L)** | 69.3 | 72.3 | 69.9 | 60.6 | 79.4 | 60.2 | 57.9 | 82.2 |
| **Fe (µg/L)** | 90 | 85 | 120 | 120 | 491.1 | 227.1 | n.a. | 90.7 |
| **Mn (µg/L)** | 395.2 | 345.6 | 261.7 | 436.8 | 378.9 | 491.1 | 537.8 | 181.3 |
| **U (µg/L)** | <0.05 | <0.05 | <0.05 | <0.05 | <0.05 | <0.05 | <0.05 | <0.05 |

Table S3 continued

| **Well ID** | NS17-Bahariya | NS18-Bahariya | NS19-Bahariya | NS20-Bahariya | NS21-Bahariya | NS22-Bahariya | NS23-Bahariya | NS24-Bahariya |
| --- | --- | --- | --- | --- | --- | --- | --- | --- |
| **TDS (mg/L)** | 162 | 615.4 | 222.6 | 146.3 | 153.9 | 182.5 | 192 | 214.6 |
| **T (°C)** | 43 | 34 | 40 | 39 | 44 | 44 | 46 | 49 |
| **pH** | 7.1 | 7 | 7.7 | 7.3 | 7 | 7.1 | 7.1 | 7.2 |
| **Ca (ppm)** | 0.9 | 5.1 | 1.3 | 0.8 | 0.9 | 1.1 | 1.2 | 1.2 |
| **Mg (ppm)** | 8.4 | 45.4 | 12.2 | 7.8 | 8 | 9.3 | 9.5 | 10.2 |
| **Na (ppm)** | 7.8 | 38.3 | 11.1 | 6.3 | 6.9 | 10.1 | 11.4 | 12.9 |
| **K (ppm)** | 13.7 | 28.5 | 19 | 11.8 | 12.6 | 15.8 | 16.3 | 18.1 |
| **F (ppm)** | 0.2 | 0.3 | 0.3 | 0.2 | 0.3 | 0.2 | 0 | 0.2 |
| **Cl (ppm)** | 43 | 198.2 | 71 | 30.5 | 38.5 | 68.3 | 67.6 | 83.4 |
| **Br (ppm)** | 0.1 | 0.2 | 0.2 | <0.1 | <0.1 | 0.2 | 0.2 | 0.2 |
| **SO_4_ (ppm)** | 15 | 211.7 | 12.4 | 19.1 | 18.7 | 10.5 | 12 | 11.6 |
| **HCO_3_ (ppm)** | 72.9 | 87.6 | 95.1 | 69.9 | 68.2 | 67.1 | 73.7 | 76.7 |
| **Ba (µg/L)** | 340.5 | 113.6 | 424.7 | 207.5 | 284.2 | 456.8 | 446.4 | 506.9 |
| **Sr (µg/L)** | 51.8 | 194 | 74.2 | 43.3 | 50.1 | 58.4 | 60.4 | 65.8 |
| **Fe (µg/L)** | 14.2 | n.a. | n.a. | n.a. | n.a. | 67.9 | 39.3 | 81.7 |
| **Mn (µg/L)** | 397.9 | 1264 | 280.6 | 517.6 | 505.8 | 563.6 | 455.9 | 478.9 |
| **U (µg/L)** | <0.05 | <0.05 | <0.05 | <0.05 | <0.05 | <0.05 | <0.05 | <0.05 |

Table S3 continued

| **Well ID** | NS25-Bahariya | NS26-Bahariya | NS27-Bahariya | NS28-Bahariya | NS29-Farafra | NS30-Farafra | NS31-Farafra | NS32-Farafra |
| --- | --- | --- | --- | --- | --- | --- | --- | --- |
| **TDS (mg/L)** | 168.3 | 234 | 282.3 | 211 | 140.4 | 107.9 | 164.7 | 156.1 |
| **T (°C)** | 48 | 34 | 37 | 46 | 35.8 | 40.4 | 38.2 | 35.8 |
| **pH** | 7.1 | 7.5 | 7.8 | 7.4 | 6.9 | 6.6 | 7 | 7 |
| **Ca (ppm)** | 1.1 | 2 | 1.6 | 1.3 | 11.2 | 9 | 10.8 | 10.8 |
| **Mg (ppm)** | 8.1 | 14.4 | 15.1 | 9.9 | 6.3 | 4.1 | 8.6 | 8.3 |
| **Na (ppm)** | 9.1 | 6.6 | 10.7 | 13.3 | 12.1 | 10.4 | 11.2 | 11.4 |
| **K (ppm)** | 14.3 | 20.2 | 25.3 | 17.8 | 9 | 3.8 | 14.8 | 12.3 |
| **F (ppm)** | 0.3 | 0.5 | 0.9 | 0.2 | 0.2 | 0.2 | 0.2 | 0.3 |
| **Cl (ppm)** | 51.7 | 36.4 | 54.2 | 77.2 | 27.5 | 19 | 23 | 24.9 |
| **Br (ppm)** | 0.1 | <0.1 | 0.2 | 0.2 | 0.2 | 0.2 | 0.2 | 0.2 |
| **SO_4_ (ppm)** | 13.7 | 24.9 | 6.9 | 11.8 | 20.8 | 17.8 | 18.4 | 18.7 |
| **HCO_3_ (ppm)** | 69.9 | 129 | 167.2 | 79.4 | 53.1 | 43.4 | 77.5 | 69.2 |
| **Ba (µg/L)** | 388.8 | 229.1 | 209.4 | 459.9 | 168.6 | 158.6 | 202.1 | 181.9 |
| **Sr (µg/L)** | 53.4 | 97.5 | 93.7 | 64.4 | 47.8 | 45.5 | 75.2 | 69 |
| **Fe (µg/L)** | n.a. | n.a. | n.a. | 263.3 | 125 | 95 | 170 | 120 |
| **Mn (µg/L)** | 538.6 | 65.1 | 35.8 | 566.1 | 295.1 | 182.5 | 185.6 | 191.9 |
| **U (µg/L)** | <0.05 | <0.05 | <0.05 | <0.05 | 0.01 | <0.05 | <0.05 | <0.05 |

Table S3 continued

| **Well ID** | NS33-Farafra | NS34-Farafra | NS35-Farafra | NS36-Farafra | NS37-Farafra | NS38-Farafra | NS39-Farafra | NS40-Farafra |
| --- | --- | --- | --- | --- | --- | --- | --- | --- |
| **TDS (mg/L)** | 156.5 | 150.1 | 142.3 | 154.8 | 142.9 | 154.5 | 126.2 | 116.5 |
| **T (°C)** | 36.2 | 35.2 | 34.6 | 35.3 | 37.7 | 36 | 40.4 | 37.4 |
| **pH** | 6.9 | 6.8 | 6.7 | 6.5 | 6.7 | 6.7 | 6.8 | 6.6 |
| **Ca (ppm)** | 10 | 10.8 | 11 | 13.2 | 13.1 | 13.9 | 10.8 | 10.5 |
| **Mg (ppm)** | 8.2 | 7.6 | 7 | 6.4 | 6.4 | 6.6 | 5.8 | 4.4 |
| **Na (ppm)** | 10.8 | 11.4 | 11.8 | 14.8 | 14.6 | 15.6 | 11.6 | 12 |
| **K (ppm)** | 13.5 | 10.8 | 9.8 | 7 | 7 | 7.8 | 7.4 | 4 |
| **F (ppm)** | 0.2 | 0.2 | 0.2 | 0.2 | 0.1 | 0.1 | 0.2 | 0.2 |
| **Cl (ppm)** | 22.8 | 26.8 | 28 | 34.8 | 34.7 | 36.6 | 25.7 | 22.1 |
| **Br (ppm)** | 0.2 | 0.2 | 0.2 | 0.1 | 0.1 | 0.1 | 0.1 | 0.1 |
| **SO_4_ (ppm)** | 19.1 | 19.8 | 20.5 | 24.1 | 23.9 | 23.6 | 20.7 | 20.6 |
| **HCO_3_ (ppm)** | 71.7 | 62.5 | 53.8 | 54.2 | 43 | 50.2 | 43.9 | 42.6 |
| **Ba (µg/L)** | 195.3 | 177.1 | 171.6 | 146.5 | 152.9 | 139.1 | 188 | 96.4 |
| **Sr (µg/L)** | 70.4 | 62.3 | 57.4 | 51.2 | 51.7 | 57.7 | 42.8 | 41.3 |
| **Fe (µg/L)** | 80 | 70 | 80 | 70 | 35 | 560 | 190 | 110 |
| **Mn (µg/L)** | 197 | 234.8 | 275.1 | 322.9 | 304.6 | 382.5 | 282.1 | 294.9 |
| **U (µg/L)** | <0.05 | <0.05 | <0.05 | <0.05 | <0.05 | <0.05 | <0.05 | <0.05 |

Table S3 continued

| **Well ID** | NS41-Farafra | NS42-Farafra | NS43-Dakhla | NS44-Dakhla | NS45-Dakhla | NS46-Dakhla | NS47-Dakhla | NS48-Dakhla |
| --- | --- | --- | --- | --- | --- | --- | --- | --- |
| **TDS (mg/L)** | 119.1 | 109 | 200 | 145.6 | 196.1 | 139.8 | 131 | 131 |
| **T (°C)** | 37.8 | 43.2 | 33.8 | 40 | 32 | 38 | 36.8 | 35.7 |
| **pH** | 6.7 | 6.6 | 6.8 | 7 | 6.8 | 7 | 7.1 | 7.1 |
| **Ca (ppm)** | 10.7 | 10.9 | 20.9 | 9.1 | 17.2 | 9.8 | 7.7 | 7.8 |
| **Mg (ppm)** | 4.3 | 3.4 | 9 | 6.3 | 8.4 | 6.9 | 6 | 6.4 |
| **Na (ppm)** | 11.6 | 12.4 | 19.4 | 6.7 | 18.1 | 7.8 | 6.7 | 6.8 |
| **K (ppm)** | 4.7 | 2.3 | 7.9 | 16.4 | 9 | 15.2 | 18.2 | 17.9 |
| **F (ppm)** | 0.2 | 0.2 | 0.1 | 0.1 | 0.1 | 0.1 | 0.2 | 0.2 |
| **Cl (ppm)** | 20.3 | 21.1 | 37.8 | 18.2 | 34.7 | 20.1 | 19 | 21.8 |
| **Br (ppm)** | 0.1 | 0.1 | 0.1 | 0.1 | 0.1 | 0.1 | 0.1 | 0.1 |
| **SO_4_ (ppm)** | 20.3 | 17.6 | 48.6 | 17.1 | 42.1 | 18.7 | 17.7 | 14.4 |
| **HCO_3_ (ppm)** | 46.9 | 41 | 56.2 | 71.6 | 66.4 | 61.1 | 55.4 | 55.6 |
| **Ba (µg/L)** | 94.4 | 56.6 | 64.4 | 230.3 | 72.4 | 202.7 | 222.2 | 268.8 |
| **Sr (µg/L)** | 39.8 | 50.6 | 98.7 | 159.6 | 86 | 149.3 | 128.6 | 138 |
| **Fe (µg/L)** | 95 | 255 | 75 | 200 | 55 | 60 | 100 | 105 |
| **Mn (µg/L)** | 293 | 117.8 | 269.5 | 186.9 | 256.7 | 126.3 | 202.4 | 256.5 |
| **U (µg/L)** | <0.05 | <0.05 | <0.05 | <0.05 | <0.05 | <0.05 | <0.05 | <0.05 |

Table S3 continued

| **Well ID** | NS49-Dakhla | NS50-Kharga | NS51-Kharga | NS52-Kharga | NS53-Kharga | NS54-Kharga | NS55-Kharga | NS56-Siwa |
| --- | --- | --- | --- | --- | --- | --- | --- | --- |
| **TDS (mg/L)** | 145.9 | 323.7 | 312.6 | 301.1 | 387.1 | 436.1 | 357.1 | 177.5 |
| **T (°C)** | 35.9 | 30.1 | 30.1 | 29.4 | 35 | 33 | 35.1 | 42 |
| **pH** | 7 | 7.3 | 7.1 | 7.2 | 7.3 | 7.5 | 7.4 | 7.54 |
| **Ca (ppm)** | 8.3 | 21.5 | 25 | 18.2 | 23.7 | 23.5 | 22.8 | 0.7 |
| **Mg (ppm)** | 7.4 | 21 | 19.9 | 16.4 | 11 | 12.2 | 10.3 | 8.1 |
| **Na (ppm)** | 7.9 | 21.4 | 20 | 20.9 | 51.3 | 67.6 | 45.8 | 8.3 |
| **K (ppm)** | 20.3 | 29.5 | 29.7 | 33.3 | 26.3 | 24.5 | 25.5 | 16.9 |
| **F (ppm)** | 0.2 | 0.7 | 0.3 | 0.7 | 0.9 | 0.8 | 0.9 | 0.4 |
| **Cl (ppm)** | 25.8 | 41.5 | 58.6 | 44.9 | 51.4 | 61.2 | 47.1 | 31.1 |
| **Br (ppm)** | 0.1 | 0.1 | 0.1 | 0.1 | 0.2 | 0.3 | 0.2 | <0.1 |
| **SO_4_ (ppm)** | 17 | 57.4 | 63.3 | 34.8 | 24.1 | 27.1 | 23.5 | 13.2 |
| **HCO_3_ (ppm)** | 58.9 | 130.6 | 95.7 | 131.8 | 198.2 | 218.9 | 181 | 98.7 |
| **Ba (µg/L)** | 304.4 | 150.6 | 86.5 | 151.7 | 223.9 | 209.7 | 193.4 | 394.1 |
| **Sr (µg/L)** | 204.4 | 173.7 | 264.2 | 160.6 | 350.7 | 407.3 | 322.5 | 31 |
| **Fe (µg/L)** | 110 | 80 | 85 | 155 | 110 | 145 | 95 | 262.5 |
| **Mn (µg/L)** | 398.8 | 329.2 | 296.2 | 208.9 | 93.3 | 84.8 | 72.7 | 114.4 |
| **U (µg/L)** | <0.05 | <0.05 | <0.05 | <0.05 | <0.05 | <0.05 | <0.05 | <0.05 |

Table S3 continued

| **Well ID** | NS57-Siwa | NS58-Siwa | NS59-Siwa | NS60-Siwa | NS61-Siwa | NS62-Siwa | NS63-Siwa | NS64-Siwa |
| --- | --- | --- | --- | --- | --- | --- | --- | --- |
| **TDS (mg/L)** | 186.3 | 218.9 | 194.7 | 222.3 | 322.9 | 194.8 | 402.3 | 195.5 |
| **T (°C)** | 42 | 42 | 39 | 40 | 41 | 41 | 39 | 42 |
| **pH** | 7.58 | 7.71 | 7.43 | 7.73 | 7.61 | 7.67 | 7.95 | 7.49 |
| **Ca (ppm)** | 0.6 | 0.7 | 0.8 | 0.9 | 1.2 | 0.9 | 3.6 | 0.9 |
| **Mg (ppm)** | 6.4 | 6 | 6.8 | 5.9 | 7.4 | 7.1 | 11.4 | 7.4 |
| **Na (ppm)** | 10.7 | 14.7 | 11.5 | 15 | 28.6 | 10.2 | 27.9 | 8.6 |
| **K (ppm)** | 15.2 | 14.4 | 17.7 | 17.2 | 20.1 | 17.5 | 22.6 | 18.4 |
| **F (ppm)** | 0.5 | 0.5 | 0.6 | 0.6 | 0.6 | 0.5 | 0.7 | 0.6 |
| **Cl (ppm)** | 30.5 | 29.1 | 32 | 34.9 | 93.6 | 29.5 | 89.3 | 27.9 |
| **Br (ppm)** | <0.1 | <0.1 | <0.1 | <0.1 | 0.2 | <0.1 | 0.1 | <0.1 |
| **SO_4_ (ppm)** | 13.7 | 13.4 | 14.8 | 15.4 | 22.5 | 13.4 | 21.8 | 12.9 |
| **HCO_3_ (ppm)** | 108.8 | 140.1 | 110.5 | 132.4 | 148.6 | 115.8 | 224.9 | 118.9 |
| **Ba (µg/L)** | 334.3 | 296.3 | 288.1 | 300.6 | 287.2 | 319.4 | 273.4 | 328.2 |
| **Sr (µg/L)** | 25.3 | 25.5 | 33 | 31.6 | 39.3 | 31.1 | 48.1 | 32.8 |
| **Fe (µg/L)** | 479.1 | 44.3 | n.a. | n.a. | n.a. | 567.9 | n.a. | 355.3 |
| **Mn (µg/L)** | 76.8 | 77.7 | 91.9 | 64.8 | 65 | 100.6 | 53.5 | 84.4 |
| **U (µg/L)** | <0.05 | <0.05 | <0.05 | <0.05 | <0.05 | <0.05 | <0.05 | <0.05 |

**Table S4. Saturation indices of common sulfate and carbonate minerals for the investigated groundwater**

|  | **Well ID** | | **Saturation Index (SI)** | | | | | | | |
| --- | --- | --- | --- | --- | --- | --- | --- | --- | --- | --- |
|  |  |  | **Calcite** | | **Barite** | | **Gypsum** | | **Celestite** | |
| NS1-Bahariya | | -0.1761 | | -0.3881 | | -3.2107 | | -4.5854 | |  |
| NS2-Bahariya | | -0.9756 | | 0.0372 | | -3.1345 | | -4.3477 | |  |
| NS3-Bahariya | | -1.5597 | | 0.2788 | | -2.3564 | | -3.7079 | |  |
| NS4-Bahariya | | -1.6302 | | 0.1463 | | -2.5654 | | -3.8387 | |  |
| NS5-Bahariya | | -1.4091 | | 0.0385 | | -3.1405 | | -4.2264 | |  |
| NS6-Bahariya | | -1.5613 | | 0.0946 | | -3.0656 | | -999.999 | |  |
| NS7-Bahariya | | -0.9668 | | 0.0334 | | -3.2168 | | -4.3832 | |  |
| NS8-Bahariya | | -1.3208 | | 0.0188 | | -3.1934 | | -4.3443 | |  |
| NS9-Bahariya | | -1.0307 | | 0.0118 | | -3.0993 | | -4.2711 | |  |
| NS10-Bahariya | | -1.7358 | | 0.1159 | | -2.6444 | | -3.8836 | |  |
| NS11-Bahariya | | -1.4831 | | 0.0043 | | -2.7442 | | -4.0126 | |  |
| NS12-Bahariya | | -1.4046 | | 0.1269 | | -3.0655 | | -4.1428 | |  |
| NS13-Bahariya | | -1.5746 | | 0.0976 | | -4.1287 | | -4.3625 | |  |
| NS14-Bahariya | | -1.9225 | | 0.1106 | | -4.2343 | | -4.3168 | |  |
| NS15-Bahariya | | -2.0227 | | 0.0444 | | -4.2039 | | -4.2864 | |  |
| NS16-Bahariya | | -1.8768 | | 0.2022 | | -3.455 | | -3.8189 | |  |
| NS17-Bahariya | | -2.0454 | | 0.0716 | | -4.1398 | | -4.1856 | |  |
| NS18-Bahariya | | -1.6263 | | 0.6168 | | -2.5293 | | -3.0695 | |  |
| NS19-Bahariya | | -1.329 | | 0.1056 | | -4.1147 | | -4.326 | |  |
| NS20-Bahariya | | -2.0581 | | 0.0612 | | -4.083 | | -999.999 | |  |
| NS21-Bahariya | | -2.1887 | | 0.1609 | | -4.038 | | -4.0815 | |  |
| NS22-Bahariya | | -2.0059 | | 0.1093 | | -4.2202 | | -4.3507 | |  |
| NS23-Bahariya | | -1.9031 | | 0.0424 | | -4.1267 | | -4.2884 | |  |
| NS24-Bahariya | | -1.7921 | | 0.0766 | | -4.15 | | -4.302 | |  |
| NS25-Bahariya | | -1.9284 | | 0.0966 | | -4.085 | | -4.2026 | |  |
| NS26-Bahariya | | -1.2626 | | 0.1788 | | -3.6463 | | -4.0617 | |  |
| NS27-Bahariya | | -0.9176 | | -0.4377 | | -4.3164 | | -4.6181 | |  |
| NS28-Bahariya | | -1.5258 | | 0.1209 | | -4.1116 | | -4.3067 | |  |
| NS29-Farafra | | -1.4318 | | 0.1254 | | -2.9167 | | -999.999 | |  |
| NS30-Farafra | | -1.8315 | | 0.0327 | | -3.0374 | | -999.999 | |  |
| NS31-Farafra | | -1.1576 | | 0.0221 | | -3.002 | | -4.142 | |  |
| NS32-Farafra | | -1.2383 | | 0.065 | | -2.9932 | | -4.1411 | |  |
| NS33-Farafra | | -1.3497 | | 0.0713 | | -3.0149 | | -4.128 | |  |
| NS34-Farafra | | -1.4885 | | 0.1033 | | -2.9635 | | -4.114 | |  |
| NS35-Farafra | | -1.6518 | | 0.1312 | | -2.9362 | | -4.0972 | |  |

Table S4 continued

| **Well ID** | **Saturation Index (SI)** | | | |
| --- | --- | --- | --- | --- |
|  | **Calcite** | **Barite** | **Gypsum** | **Celestite** |
| NS36-Farafra | -1.7654 | -0.1167 | -2.7946 | -4.0332 |
| NS37-Farafra | -1.6315 | 0.153 | -2.7935 | -4.0226 |
| NS38-Farafra | -1.5685 | -0.139 | -2.7836 | -4.0429 |
| NS39-Farafra | -1.5595 | 0.0733 | -2.9183 | -999.999 |
| NS40-Farafra | -1.8217 | -0.1784 | -2.9239 | -999.999 |
| NS41-Farafra | -1.6667 | -0.1902 | -2.9221 | -999.999 |
| NS42-Farafra | -1.7354 | -0.3081 | -2.955 | -4.0867 |
| NS43-Dakhla | -1.3011 | 0.1639 | -2.3438 | -3.789 |
| NS44-Dakhla | -1.2298 | -0.0074 | -3.0801 | -3.8393 |
| NS45-Dakhla | -1.3333 | 0.1385 | -2.4801 | -3.8444 |
| NS46-Dakhla | -1.2952 | 0.0531 | -3.0154 | -4.115 |
| NS47-Dakhla | -1.3538 | 0.0579 | -3.1322 | -4.1308 |
| NS48-Dakhla | -1.3615 | 0.1563 | -3.2188 | -4.226 |
| NS49-Dakhla | -1.414 | 0.2116 | -3.1344 | -3.8672 |
| NS50-Kharga | -0.5157 | 0.516 | -2.3417 | -3.504 |
| NS51-Kharga | -0.7834 | 0.2569 | -2.2327 | -3.2881 |
| NS52-Kharga | -0.6777 | 0.3346 | -2.6008 | -3.6906 |
| NS53-Kharga | -0.2206 | 0.0819 | -2.656 | -3.5369 |
| NS54-Kharga | -0.0243 | 0.1431 | -2.631 | -3.5123 |
| NS55-Kharga | -0.1695 | 0.0811 | -2.6717 | -3.661 |
| NS56-Siwa | -1.6451 | 0.1518 | -4.311 | -999.999 |
| NS57-Siwa | -1.632 | 0.0499 | -4.3568 | -999.999 |
| NS58-Siwa | -1.3376 | 0.0307 | -4.3138 | -999.999 |
| NS59-Siwa | -1.6924 | 0.1146 | -4.2082 | -999.999 |
| NS60-Siwa | -1.2624 | 0.113 | -4.1497 | -999.999 |
| NS61-Siwa | -1.2208 | 0.217 | -3.9076 | -999.999 |
| NS62-Siwa | -1.3584 | 0.0446 | -4.201 | -999.999 |
| NS63-Siwa | -0.2891 | 0.1877 | -3.5009 | -999.999 |
| NS64-Siwa | -1.5093 | 0.0152 | -4.2152 | -999.999 |

**Table S5. Annual radiation dose rates for groundwater wells for different life ages of population; including infants (<1 year), children (1–15 years) and adults (> 15 years)**

| **Well ID** | Annual Radiation Dose mSv/yr | | | | | |
| --- | --- | --- | --- | --- | --- | --- |
|  | infant | 1 year | 5 years | 10 years | 15 years | adult |
| NS1-Bahariya | 0.96 | 0.18 | 0.22 | 0.25 | 0.35 | 0.09 |
| NS2-Bahariya | 3.78 | 0.71 | 0.85 | 0.99 | 1.39 | 0.38 |
| NS3-Bahariya | 0.19 | 0.04 | 0.04 | 0.05 | 0.07 | 0.02 |
| NS4-Bahariya | 0.29 | 0.05 | 0.06 | 0.08 | 0.11 | 0.03 |
| NS5-Bahariya | 13.77 | 2.58 | 3.11 | 3.61 | 5.13 | 1.43 |
| NS6-Bahariya | 7.77 | 1.46 | 1.76 | 2.04 | 2.87 | 0.79 |
| NS7-Bahariya | 6.34 | 1.19 | 1.43 | 1.66 | 2.36 | 0.66 |
| NS8-Bahariya | 6.92 | 1.3 | 1.56 | 1.81 | 2.57 | 0.71 |
| NS9-Bahariya | 13.18 | 2.48 | 2.98 | 3.45 | 4.86 | 1.34 |
| NS10-Bahariya | 0.27 | 0.05 | 0.06 | 0.07 | 0.1 | 0.03 |
| NS11-Bahariya | 0.36 | 0.07 | 0.08 | 0.09 | 0.13 | 0.04 |
| NS12-Bahariya | 8.78 | 1.66 | 1.99 | 2.29 | 3.19 | 0.86 |
| NS13-Bahariya | 3.75 | 0.7 | 0.85 | 0.98 | 1.4 | 0.39 |
| NS14-Bahariya | 4.94 | 0.93 | 1.12 | 1.29 | 1.82 | 0.5 |
| NS15-Bahariya | 4.92 | 0.93 | 1.11 | 1.29 | 1.79 | 0.49 |
| NS16-Bahariya | 0.36 | 0.07 | 0.08 | 0.09 | 0.13 | 0.04 |
| NS17-Bahariya | 12.43 | 2.31 | 2.8 | 3.28 | 4.77 | 1.37 |
| NS18-Bahariya | 1.1 | 0.21 | 0.25 | 0.29 | 0.4 | 0.11 |
| NS19-Bahariya | 4.72 | 0.89 | 1.07 | 1.23 | 1.72 | 0.47 |
| NS20-Bahariya | 8.88 | 1.67 | 2.01 | 2.33 | 3.29 | 0.91 |
| NS21-Bahariya | 13.5 | 2.53 | 3.05 | 3.54 | 5.03 | 1.4 |
| NS22-Bahariya | 6.41 | 1.2 | 1.45 | 1.68 | 2.39 | 0.67 |

Table S5 continued

| **Well ID** | Annual Radiation Dose mSv/yr | | | | | |
| --- | --- | --- | --- | --- | --- | --- |
|  | infant | 1 year | 5 years | 10 years | 15 years | adult |
| NS23-Bahariya | 4.45 | 0.84 | 1 | 1.16 | 1.63 | 0.45 |
| NS24-Bahariya | 5 | 0.94 | 1.13 | 1.31 | 1.83 | 0.5 |
| NS25-Bahariya | 10.89 | 2.06 | 2.46 | 2.84 | 3.95 | 1.06 |
| NS26-Bahariya | 0.8 | 0.15 | 0.18 | 0.21 | 0.29 | 0.08 |
| NS27-Bahariya | 1.07 | 0.2 | 0.24 | 0.28 | 0.4 | 0.11 |
| NS28-Bahariya | 5.06 | 0.96 | 1.15 | 1.32 | 1.84 | 0.5 |
| min | 0.19 | 0.04 | 0.04 | 0.05 | 0.07 | 0.02 |
| max | 13.77 | 2.58 | 3.11 | 3.61 | 5.13 | 1.43 |
| Median | 4.93 | 0.93 | 1.11 | 1.29 | 1.81 | 0.49 |
| NS29-Farafra | 2.08 | 0.39 | 0.47 | 0.54 | 0.76 | 0.21 |
| NS30-Farafra | 2.61 | 0.49 | 0.59 | 0.68 | 0.98 | 0.28 |
| NS31-Farafra | 1.04 | 0.2 | 0.23 | 0.27 | 0.39 | 0.11 |
| NS32-Farafra | 1.31 | 0.25 | 0.3 | 0.34 | 0.49 | 0.13 |
| NS33-Farafra | 1.53 | 0.29 | 0.35 | 0.4 | 0.56 | 0.15 |
| NS34-Farafra | 1.36 | 0.26 | 0.31 | 0.35 | 0.5 | 0.14 |
| NS35-Farafra | 0.85 | 0.16 | 0.19 | 0.22 | 0.31 | 0.09 |
| NS36-Farafra | 2.53 | 0.48 | 0.57 | 0.66 | 0.92 | 0.25 |
| NS37-Farafra | 1.68 | 0.32 | 0.38 | 0.44 | 0.61 | 0.17 |
| NS38-Farafra | 1.6 | 0.3 | 0.36 | 0.42 | 0.59 | 0.16 |
| NS39-Farafra | 1.76 | 0.33 | 0.4 | 0.46 | 0.65 | 0.18 |
| NS40-Farafra | 1.58 | 0.3 | 0.36 | 0.41 | 0.58 | 0.16 |
| NS41-Farafra | 1.47 | 0.28 | 0.33 | 0.39 | 0.54 | 0.15 |

Table S5 continued

| **Well ID** | Annual Radiation Dose mSv/yr | | | | | |
| --- | --- | --- | --- | --- | --- | --- |
|  | infant | 1 year | 5 years | 10 years | 15 years | adult |
| NS42-Farafra | 1.72 | 0.32 | 0.39 | 0.45 | 0.64 | 0.18 |
| min | 0.85 | 0.16 | 0.19 | 0.22 | 0.31 | 0.09 |
| max | 2.61 | 0.49 | 0.59 | 0.68 | 0.98 | 0.28 |
| Median | 1.59 | 0.3 | 0.36 | 0.42 | 0.58 | 0.16 |
| NS43-Dakhla | 1.47 | 0.28 | 0.33 | 0.39 | 0.54 | 0.15 |
| NS44-Dakhla | 2.71 | 0.51 | 0.61 | 0.71 | 1 | 0.27 |
| NS45-Dakhla | 0.94 | 0.18 | 0.21 | 0.25 | 0.34 | 0.09 |
| NS46-Dakhla | 1.93 | 0.37 | 0.44 | 0.5 | 0.7 | 0.19 |
| NS47-Dakhla | 2.09 | 0.4 | 0.47 | 0.55 | 0.76 | 0.2 |
| NS48-Dakhla | 2.94 | 0.56 | 0.67 | 0.77 | 1.07 | 0.29 |
| NS49-Dakhla | 1.86 | 0.35 | 0.42 | 0.49 | 0.7 | 0.2 |
| min | 0.94 | 0.18 | 0.21 | 0.25 | 0.34 | 0.09 |
| max | 2.94 | 0.56 | 0.67 | 0.77 | 1.07 | 0.29 |
| Median | 1.93 | 0.37 | 0.44 | 0.5 | 0.7 | 0.2 |
| NS50-Kharga | 0.64 | 0.12 | 0.14 | 0.17 | 0.24 | 0.07 |
| NS51-Kharga | 0.67 | 0.13 | 0.15 | 0.18 | 0.25 | 0.07 |
| NS52-Kharga | 0.97 | 0.18 | 0.22 | 0.25 | 0.36 | 0.1 |
| NS53-Kharga | 3.11 | 0.58 | 0.7 | 0.81 | 1.15 | 0.32 |
| NS54-Kharga | 2.62 | 0.49 | 0.59 | 0.69 | 0.97 | 0.27 |
| NS55-Kharga | 2.31 | 0.43 | 0.52 | 0.61 | 0.86 | 0.24 |
| min | 0.64 | 0.12 | 0.14 | 0.17 | 0.24 | 0.07 |
| max | 3.11 | 0.58 | 0.7 | 0.81 | 1.15 | 0.32 |
| Median | 1.64 | 0.31 | 0.37 | 0.43 | 0.61 | 0.17 |

Table S5 continued

| **Well ID** | Annual Radiation Dose mSv/yr | | | | | |
| --- | --- | --- | --- | --- | --- | --- |
|  | infant | 1 year | 5 years | 10 years | 15 years | adult |
| NS56-Siwa | 0.75 | 0.14 | 0.17 | 0.2 | 0.28 | 0.08 |
| NS57-Siwa | 0.74 | 0.14 | 0.17 | 0.19 | 0.28 | 0.08 |
| NS58-Siwa | 0.56 | 0.1 | 0.13 | 0.15 | 0.21 | 0.06 |
| NS59-Siwa | 1.08 | 0.2 | 0.24 | 0.28 | 0.4 | 0.11 |
| NS60-Siwa | 0.61 | 0.11 | 0.14 | 0.16 | 0.23 | 0.06 |
| NS61-Siwa | 0.66 | 0.12 | 0.15 | 0.17 | 0.25 | 0.07 |
| NS62-Siwa | 0.89 | 0.17 | 0.2 | 0.23 | 0.33 | 0.09 |
| NS63-Siwa | 1.44 | 0.27 | 0.32 | 0.38 | 0.53 | 0.15 |
| NS64-Siwa | 0.8 | 0.15 | 0.18 | 0.21 | 0.31 | 0.09 |
| min | 0.56 | 0.1 | 0.13 | 0.15 | 0.21 | 0.06 |
| max | 1.44 | 0.27 | 0.32 | 0.38 | 0.53 | 0.15 |
| Median | 0.75 | 0.14 | 0.17 | 0.2 | 0.28 | 0.08 |

**References**

1. Szabo, Z., Depaul, V. T., Fischer, J. M., Kraemer, T. F. & Jacobsen, E. Occurrence and geochemistry of radium in water from principal drinking-water aquifer systems of the United States. *Applied Geochemistry* **27,** 729–752 (2012).
2. Sherif, M. I. & Sturchio, N. C. Radionuclide geochemistry of groundwater in the Eastern Desert, Egypt. *Applied Geochemistry* **93,** 69–80 (2018).
3. Sherif, M. I. *et al.* Geological and hydrogeochemical controls on radium isotopes in groundwater of the Sinai Peninsula, Egypt. *Science of The Total Environment* **613-614,** 877–885 (2018).
4. Vengosh, A. *et al.* High Naturally Occurring Radioactivity in Fossil Groundwater from the Middle East. *Environmental Science & Technology* **43,** 1769–1775 (2009).
5. Almasoud, F. I., Ababneh, Z. Q., Alanazi, Y. J., Khandaker, M. U. & Sayyed, M. I. Assessment of radioactivity contents in bedrock groundwater samples from the northern region of Saudi Arabia. *Chemosphere* **242**, 125181 (2020).
6. Lively, R.S., Jameson, R., Alexander, E.C., and Morey, G.B. Radium in the Mt. Simon‐Hinckley aquifer, east‐central and southeastern Minnesota: Minnesota Geological Survey, Information Circular 36 (1992).
7. Grundl, T. & Cape, M. Geochemical Factors Controlling Radium Activity in a Sandstone Aquifer. *Ground Water* **44,** 518–527 (2006).
8. Weaver, T. R. & Bahr, J. Geochemical Evolution in the Cambrian-Ordovician Sandstone Aquifer, Eastern Wisconsin: 1. Major Ion and Radionuclide Distribution. *Ground Water* **29,** 350–356 (1991).
9. Dickson, B., Giblin, A. & Snelling, A. The source of radium in anomalous accumulations near sandstone escarpments, Australia. *Applied Geochemistry* **2,** 385–398 (1987).
10. Elliot, T., Bonotto, D. M. & Andrews, J. N. Dissolved uranium, radium and radon evolution in the Continental Intercalaire aquifer, Algeria and Tunisia. *Journal of Environmental Radioactivity* **137,** 150–162 (2014).
11. Zhuo W, Iida T, Yang X. Occurrence of ^222^Rn, ^226^Ra, ^228^Ra and U in groundwater in Fujian Province, China. *Journal of Environmental Radioactivity* **53**, 111–120 (2001). doi:10.1016/S0265-931X(00)00108-9
